# Development and Functionalization of Visible-Light-Driven Water-Splitting Photocatalysts

**DOI:** 10.3390/nano12030344

**Published:** 2022-01-21

**Authors:** Tokuhisa Kawawaki, Masanobu Kawachi, Daichi Yazaki, Yuki Akinaga, Daisuke Hirayama, Yuichi Negishi

**Affiliations:** 1 Department of Applied Chemistry, Faculty of Science, Tokyo University of Science, Kagurazaka, Shinjuku-ku, Tokyo 162-8601, Japan; kawawaki@rs.tus.ac.jp (T.K.); iwish117@outlook.jp (M.K.); dunkshot1997.tus.ok@gmail.com (D.Y.); yuki.tennis.k.1214@gmail.com (Y.A.); d24.bsk5@gmail.com (D.H.); 2 Research Institute for Science & Technology, Tokyo University of Science, Kagurazaka, Shinjuku-ku, Tokyo 162-8601, Japan; 3 Center for Space System Innovation, Tokyo University of Science, Yamazaki, Noda, Chiba 278-8510, Japan

**Keywords:** visible-light-driven photocatalyst, cocatalyst, water splitting, carbon neutral, metal nanocluster, nanoparticle, functionalization

## Abstract

With global warming and the depletion of fossil resources, our fossil fuel-dependent society is expected to shift to one that instead uses hydrogen (H_2_) as a clean and renewable energy. To realize this, the photocatalytic water-splitting reaction, which produces H_2_ from water and solar energy through photocatalysis, has attracted much attention. However, for practical use, the functionality of water-splitting photocatalysts must be further improved to efficiently absorb visible (Vis) light, which accounts for the majority of sunlight. Considering the mechanism of water-splitting photocatalysis, researchers in the various fields must be employed in this type of study to achieve this. However, for researchers in fields other than catalytic chemistry, ceramic (semiconductor) materials chemistry, and electrochemistry to participate in this field, new reviews that summarize previous reports on water-splitting photocatalysis seem to be needed. Therefore, in this review, we summarize recent studies on the development and functionalization of Vis-light-driven water-splitting photocatalysts. Through this summary, we aim to share current technology and future challenges with readers in the various fields and help expedite the practical application of Vis-light-driven water-splitting photocatalysts.

## 1. Introduction

### 1.1. Water-Splitting Photocatalysts

**Figure 1:**
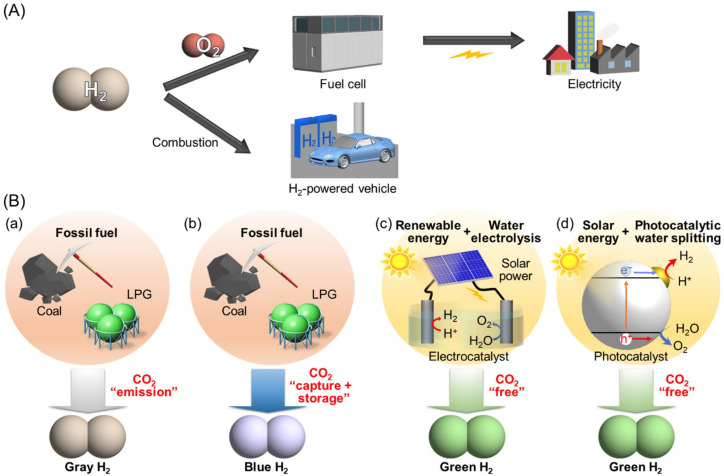
(**A**) Schematic of the expected use of H_2_ in a “H_2_-energy society”. (**B**) Coloration of H_2_ production methods: (**a**) gray H_2_, (**b**) blue H_2_, and (**c**) green H_2_ produced by renewable energy, and (**d**) green H_2_ produced by water-splitting photocatalyst. LPG represents liquefied petroleum gas.

With the increasing threat of global warming and the depletion of fossil resources, society is expected to shift to using clean and renewable energy instead of fossil fuels. Hydrogen (H_2_) does not emit carbon dioxide or other harmful materials when used in energy generation. In addition to producing energy in combustion engines, H_2_ can also be directly converted into electric power by fuel cells (Figure 1A). If H_2_ can be produced via a photocatalytic water-splitting reaction [1,2,3,4,5,6,7,8,9,10,11,12,13,14,15,16,17,18,19,20,21,22,23,24,25,26,27,28,29,30,31,32,33,34,35,36,37,38,39,40,41,42,43], renewable energy can be produced from sunlight and water, which are abundant on Earth (Figure 1B). Therefore, water-splitting photocatalysts have attracted huge attention as a means to address energy and environmental problems.

**Figure 2:**
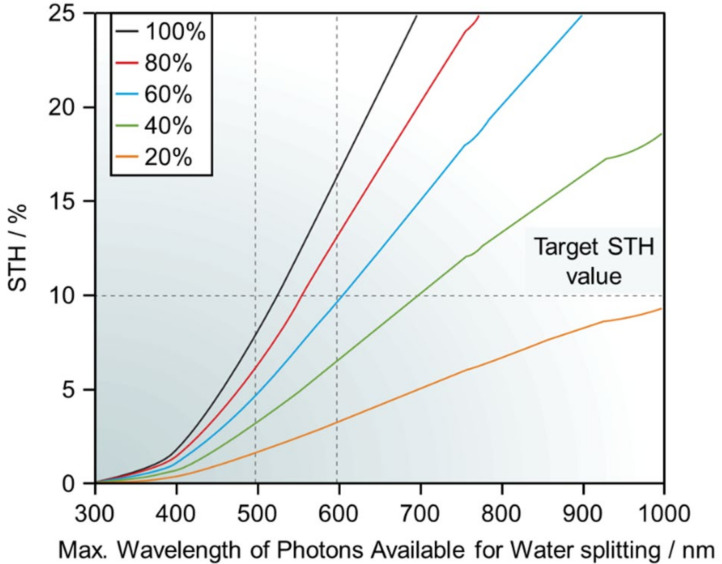
Different apparent quantum yields (AQYs) covering the typical range of 20–100%. Dashed lines represent STH efficiencies at different AQYs with the maximum operable wavelength of 500 or 600 nm and the target STH value of 10%. Associated calculations assumed AM1.5G solar irradiance. Reproduced with permission from reference [44]. Copyright 2017 Springer Nature Limited.

However, to realize the practical use of water-splitting photocatalysts, solar-to-hydrogen conversion efficiency (STH) should be improved to about 10%. As shown in Figure 2, it is difficult to achieve STH = 10% using only ultraviolet (UV) light [44]. Therefore, the use of visible (Vis)-light-driven water-splitting photocatalysts that conduct water splitting under Vis light (400 nm ≤ λ ≤ 800 nm), which accounts for most sunlight, is essential to achieve this goal [45,46,47,48].

### 1.2. Development of Vis-Light-Driven Water-Splitting Photocatalysts

**Figure 3:**
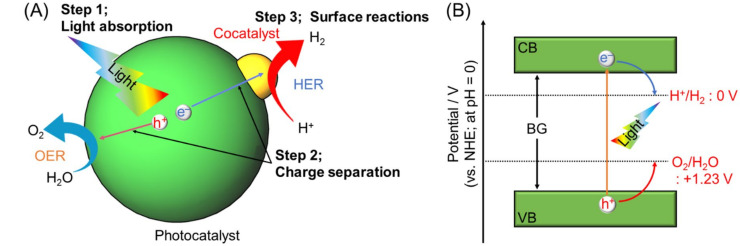
(**A**) Schematic of photocatalytic water-splitting reactions: Step 1, light absorption; Step 2, charge separation; and Step 3, surface reactions. (**B**) Principle of water splitting using semiconductor photocatalysts. HER, OER, VB CB, and BG represent hydrogen evolution reaction, oxygen evolution reaction, valence band, conduction band, and band gap, respectively.

When a water-splitting reaction is conducted using a semiconductor photocatalyst, the reaction consists of three main steps (Figure 3A), as follows: (1) The semiconductor photocatalyst absorbs light energy, resulting in electronic excitation from the valence band (VB) to the conduction band (CB); (2) the excited electrons and remaining holes in the VB migrate to the photocatalyst surface or cocatalyst nanoparticles (NPs), respectively; (3) the H_2_ evolution reaction (HER; Equation (1)) and oxygen (O_2_) evolution reaction (OER; Equation (2)) proceed on the cocatalyst or photocatalyst surface.

2H^+^ + 2e^−^ → H_2_ (1)

2H_2_O + 4h^+^ → 4H^+^ + O_2_ (2)

**Figure 4:**
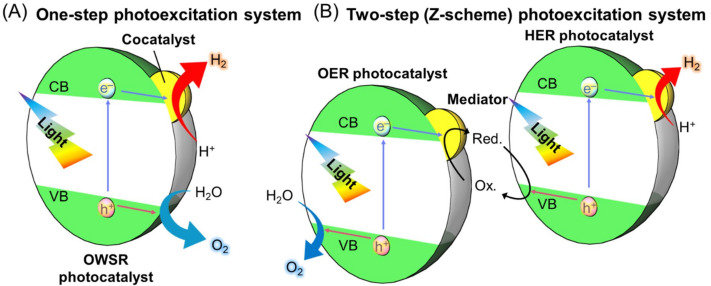
Schematic illustrations of photocatalytic reactions: (**A**) one-step photoexcitation system for overall water-splitting reaction (OWSR); (**B**) two-step (Z-scheme) photoexcitation system for OWSR. Red. and Ox. represent reductants and oxidants, respectively.

Theoretically, H_2_O reduction and H_2_ evolution proceed when the CB minimum edge (CBM) of the semiconductor photocatalyst is more negative than the reduction potential of H_2_O (0 V vs. normal H_2_ electrode (NHE); pH = 0). The H_2_O oxidation reaction proceeds, and O_2_ is evolved when the VB maximum edge (VBM) of the semiconductor photocatalyst is more positive than the oxidation potential of H_2_O (1.23 V vs. NHE; pH = 0) (Figure 3B). When the band gap (BG) of the semiconductor photocatalyst is sufficiently wide (UV-light-driven photocatalyst), such control of the CBM and VBM positions is relatively easy. In contrast, when the BG of the semiconductor photocatalyst is narrow (Vis-light-driven water-splitting photocatalyst), achieving appropriate CBM and VBM positions simultaneously becomes difficult. Furthermore, a semiconductor photocatalyst with a CBM and VBM that satisfy the above conditions is not guaranteed to achieve an overall water-splitting reaction (OWSR). This is due to the following factors: (i) The high activation energy of the water-splitting reaction makes it difficult for the reaction to proceed; (ii) recombination of electrons and holes (excitons) causes the reaction to be deactivated; and (iii) generated H_2_ and O_2_ cause a reverse reaction. Therefore, only a few studies have reported one-step photocatalytic materials that can achieve an OWSR under Vis light (Figure 4A) [49].

The OWSR can also be achieved by combining two semiconductor photocatalysts, which can conduct the half-reactions of water splitting (HER and OER), and a redox couple (mediator), which can transfer excitons between them (Figure 4B) [50,51]. This two-step reaction system, which imitates plant photosynthesis, is called the Z-scheme water-splitting reaction. In this reaction, any semiconductor that can cause a half-reaction can be used. Therefore, the number of available photocatalysts in this reaction is much larger than in the one-step water-splitting reaction, and longer-wavelength light can be used. As the HER and OER occur on two separate photocatalysts, the reverse reaction of H_2_ and O_2_ evolution can be suppressed in the Z-scheme system using a two-port H-type electrolytic cell with an ion-exchange membrane. Furthermore, separation of the evolved gases is not necessary using this method. However, the reverse reaction involving the redox couples can occur in this system, which does not occur in the one-step water-splitting reaction [51]. Furthermore, as a disadvantage, the theoretical STH of the Z-scheme water-splitting reaction is lower than that of the one-step water-splitting reaction, as two photons are required in a single reaction.

Unfortunately, at present, there is no Vis-light-driven water-splitting photocatalyst that can realize practical application in either the one-step or Z-scheme reaction. To generate sufficient H_2_ to withstand market competition using water-splitting photocatalysts, and thereby realize a H_2_-energy society, it is essential to greatly improve the functionality of Vis-light-driven photocatalysts in the future.

### 1.3. Purpose of This Review

Considering the mechanism of water-splitting photocatalysis (Figure 3), researchers in the fields of catalytic chemistry, ceramic (semiconductor) materials chemistry, electrochemistry, metal NP/nanocluster (NC) chemistry [52,53,54,55,56,57,58,59,60,61,62,63,64,65,66,67,68], surface spectroscopy [69,70,71], and theoretical chemistry [72] must be employed to create highly functional Vis-light-driven water-splitting photocatalysts. Actually, we specialize in the chemical composition/structure control of metal NCs and have succeeded in enhancing the functionality of some UV-light-driven water-splitting photocatalysts by applying these techniques to water-splitting photocatalysts [73,74,75,76,77,78,79]. However, for researchers in metal NP/NC chemistry, surface spectroscopy, and theoretical chemistry, among other fields, to participate in this field, new reviews that summarize previous reports on water-splitting photocatalysis seem to be needed. Therefore, this review summarizes recent reports on the development and functionalization of Vis-light-driven water-splitting photocatalysts, and future issues are discussed. Through this summary, we aim to share the current technology and future issues with readers not previously involved in water-splitting photocatalysis and help expedite the practical application of Vis-light-driven water-splitting photocatalysts.

### 1.4. Structure of This Review

**Figure 5:**
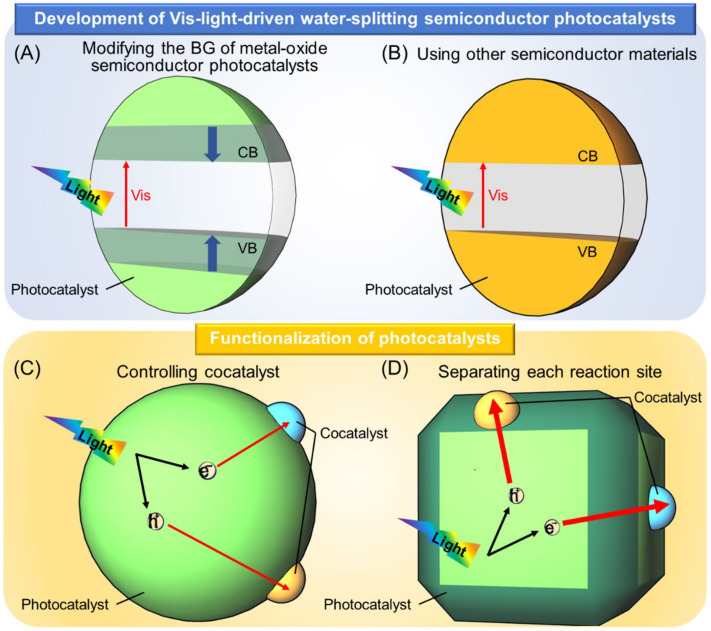
Schematic illustrations of the contents of Section 2, Section 3 and Section 4 in this review. Creation of Vis-light-driven water-splitting semiconductor photocatalysts by (**A**) modifying the BG of metal oxide semiconductor photocatalysts (UV-light-driven water-splitting photocatalysts; Section 2.1) and (**B**) using other semiconductor materials (Section 2.2). Functionalization of photocatalysts by (**C**) controlling the cocatalysts (Section 3) and (**D**) separating each reaction site (Section 4).

The outline of this review is as follows. Section 2 describes the methods used to develop Vis-light-driven water-splitting photocatalysts. Specifically, Section 2.1 describes the fabrication of Vis-light-driven water-splitting photocatalysts by modifying the BG of metal oxide water-splitting photocatalysts (UV-light-driven water-splitting photocatalyst) (Figure 5A), and Section 2.2 describes the development of Vis-light-driven water-splitting photocatalysts using materials other than metal oxides (Figure 5B). Section 3 describes controlling the cocatalyst (Figure 3A), which operates as the active site (Figure 5C). Section 4 describes means for separating each reaction site (Figure 5D) to enhance the efficiency of the water-splitting reaction. Section 5 provides a short summary and, finally, Section 6 describes our future outlook.

In this review, we have categorized methods for the fabrication and functionalization of Vis-light-driven water-splitting photocatalysts according to our viewpoint. Therefore, this categorization might differ from previous reviews [44,49,51,80,81,82,83,84,85,86,87,88,89,90]. Furthermore, as this review was written for readers not previously involved in water-splitting photocatalysis, methods used to prepare each photocatalyst and analyze their properties are not described. Therefore, readers interested in details of these experiments are referred to the original papers cited.

## 2. Creation of Vis-Light-Driven Water-Splitting Semiconductor Photocatalysts

### 2.1. Modification of BG of Metal Oxide Semiconductor Photocatalysts (UV-Light-Driven Water-Splitting Photocatalysts)

As metals form strong bonds with O, metal oxides are highly stable. Therefore, early studies on water-splitting semiconductor photocatalysts have mainly used metal oxides as photocatalytic materials. Such research has shown that metal oxides with an electronic structure of d^0^ (transition metal ions, such as Ti^4+^, zirconium ion (Zr^4+^), niobium ion (Nb^5+^), tantalum ion (Ta^5+^), vanadium ion (V^5+^), tungsten ion (W^6+^), and cerium ion (Ce^4+^)) and d^10^ (typical metal ions, such as zinc ion (Zn^2+^), indium ion (In^3+^), gallium ion (Ga^3+^), germanium ion (Ge^4+^), tin ion (Sn^4+^), and antimony ion (Sb^5+^)), possess functions as water-splitting photocatalysts. However, as most of these metal oxides have a BG of more than 3 eV, they can cause an OWSR only when irradiated with UV light. Therefore, to cause a water-splitting reaction under Vis light, the BG of the semiconductor photocatalyst must be narrowed to match the energy of the Vis-light region (BG < 3.0 eV).

**Figure 6:**
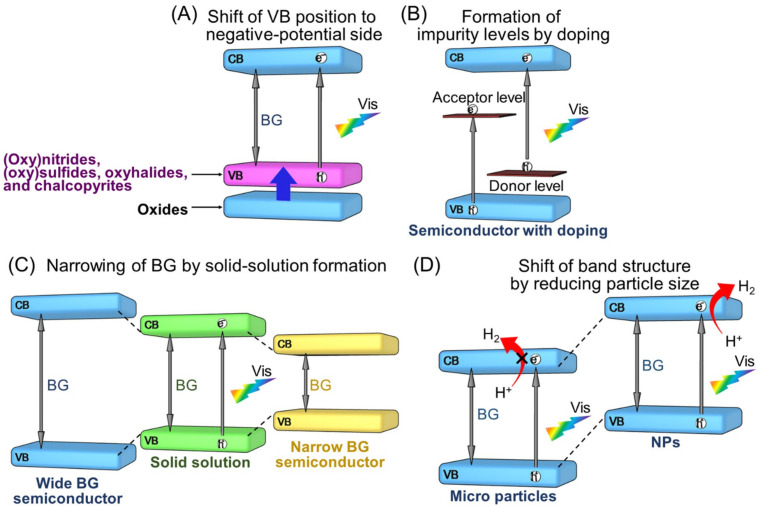
Schematic illustrations showing strategies for obtaining Vis-light-driven water-splitting photocatalysts. (**A**) Shift of VB position to negative-potential side, (**B**) formation of impurity levels by doping, (**C**) narrowing of BG by solid-solution formation, and (**D**) shift of band structure by reducing particle size.

**Figure 7:**
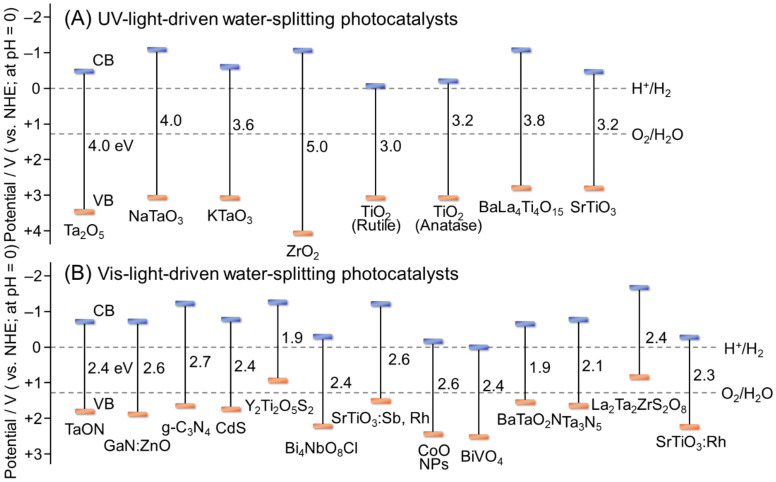
Band structures of (**A**) UV-light-driven and (**B**) Vis-light-driven water-splitting photocatalysts. In this figure, only semiconductors for which the band structure at pH = 0 has been reported are described. For the BG values of these and other semiconductors, please also see Table 1 [51,87,90,91,92,93,94,95,96,97,98,99].

The VB of metal oxide semiconductor photocatalysts consists of O 2p orbitals, with the VBM located at approx. +3 V (vs. NHE; at pH = 0) in many cases [91]. Four methods have been reported for modifying electronic structures to cause these semiconductor photocatalysts to absorb Vis light, as follows: (i) shifting the energy position of the VBM to the negative-potential side by substituting anions or metal cations (Section 2.1.1; Figure 6A) [92,93,94,95,96,97,98,99,100,101,102,103,104,105,106,107,108,109,110,111,112,113,114,115,116,117,118]; (ii) formation of impurity levels in the BG by doping (Section 2.1.2; Figure 6B) [119,120,121,122,123,124,125,126,127,128]; (iii) narrowing the BG itself through solid-solution formation (Section 2.1.3; Figure 6C) [129,130,131,132,133]; and (iv) shifting the whole band structure to the negative-potential side by reducing particle size (Section 2.1.4; Figure 6D) [134,135]. In this section, typical studies using each of these four methods are described. The BG energy, type and loading amount of appropriate cocatalysts, possible reactions (OWSR, HER, or OER; Figure 4), activity, and references for semiconductor photocatalysts described in this section are summarized in Figure 7 and Table 1 [51,87,90,91,92,93,94,95,96,97,98,99,100,101,102,103,105,106,107,109,110,112,114,116,117,118,119,120,128,129,130,131,132,134].

#### 2.1.1. Shift of VB Position to Negative-Potential Side

##### Substitution of O^2−^ Anion by Nitrogen, Sulfur, or Halide Anion

When the O^2−^ of a metal oxide is substituted by a nitrogen anion (N^3−^), sulfur anion (S^2−^), halide anion (chloride anion (Cl^−^), bromide anion (Br^−^), or iodide anion (I^−^)), a new VB can be constructed on the negative-potential side of the O 2p orbital of the metal oxide.

**Figure 8:**
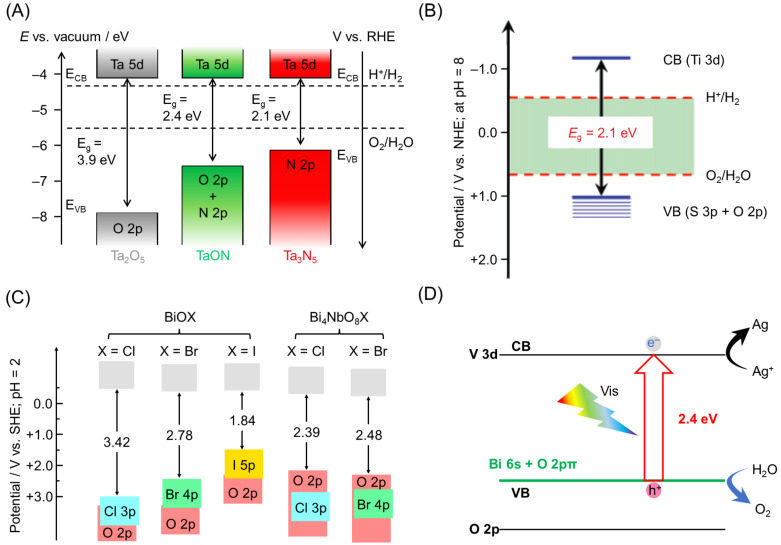
(**A**) Band diagrams for Ta_2_O_5_, TaON, and Ta_3_N_5_ showing the shift in VBM upon nitridation. RHE represents the reversible hydrogen electrode. (**B**) Schematic illustration of band edge potentials of Sm_2_Ti_2_S_2_O_5_. (**C**) Schematic illustration of VB and CB structures of BiOX (X = Cl, Br, or I) and Bi_4_NbO_8_X (X = Cl or Br). Red, blue, green, and orange boxes represent the bands from O 2p, Cl 3p, Br 4p, and I 5p orbitals, respectively, and the gray box shows a CB composed mainly of Bi 6p. (**D**) Band structures of *m*-BiVO_4_. Reproduced with permission from references [86,92,99,109]. Copyright 2015 American Chemical Society, 2007 American Chemical Society, 2017 American Chemical Society, and 2009 The Royal Society of Chemistry.

Figure 8A shows the band structure of metal oxide Ta_2_O_5_ and TaON, whose O^2−^ were substituted by N^3−^ (Figure 7 and Table 1) [92]. The VBM of Ta_2_O_5_ consists of O 2p orbitals and is located at +3.4 V (vs. NHE; at pH = 0). In contrast, in TaON, N 2p orbitals also contribute to VB formation, shifting the VBM to +2.1 V (vs. NHE; at pH = 0). The VBM shifts further to +1.7 V (vs. NHE; at pH = 0) when Ta_3_N_5_ without O is allowed to form. TaON and Ta_3_N_5_ can absorb light up to about 520 and 600 nm, respectively, and are capable of the OWSR under Vis-light irradiation (Figure 7 and Table 1) [93].

Meanwhile, the metal oxysulfide photocatalyst substituted with S^2−^ (Figure 7 and Table 1) has a VBM located on the negative-energy side compared with the metal oxide because the VB is formed by the hybridization of S 3p and O 2p orbitals (Figure 8B) [99,100,101,102,103,104,105]. For example, the VBMs of La_2_Ta_2_ZrS_2_O_8_ and Y_2_Ti_2_O_5_S_2_ are located at +0.84 V (vs. NHE; at pH = 0) and +0.8–0.9 V (vs. NHE; at pH = 9), respectively [102,103]. Regarding metal sulfide photocatalysts, Domen and co-workers and Chen and co-workers reported that the VBM can be further raised by doping with selenium (Se) or phosphorus (P), respectively [106,107].

Furthermore, in recent years, several examples of the VB being shifted to the negative-potential side by substituting O^2−^ with halide ions have been reported (Table 1) [108,109,110,111]. In such cases, the VBM shifts continuously to the negative-potential side with increasing electronegativity of the halide anion. For example, in bismuth oxyhalide (BiOX), the BGs of BiOCl, BiOBr, and BiOI are 3.42, 2.78, and 1.84 eV, respectively (Figure 8C) [108,109]. In these metal halide photocatalysts, the Bi 6p orbital forms a CBM at a more positive position compared with the reduction potential of H_2_O. Therefore, this photocatalyst alone cannot conduct water-splitting reactions under Vis-light irradiation. Accordingly, metal halide photocatalysts are expected to be OER photocatalysts in the Z-scheme. Regarding such metal halide photocatalysts, in 2016, Kageyama, Abe, and co-workers reported that the formation of Bi_4_NbO_8_Cl with Nb oxide enhanced its stability under Vis-light irradiation [109,110].

##### Substitution of Metal Cation

The BG of the semiconductor can also be narrowed by introducing metal ions with a d^10^s^2^-type electron configuration, such as copper ion (Cu^+^), silver ion (Ag^+^), lead ion (Pb^2+^), and Bi^3+^, into the metal oxide photocatalyst, resulting in a widened VB through hybridization of their orbitals with O 2p orbitals [112,113,114,115,116,117,118]. For example, in 1999, Kudo and co-workers developed monoclinic BiVO_4_ (*m*-BiVO_4_) by adding Bi^3+^ to V oxide and found that the BG of this photocatalyst was narrowed to 2.4 eV owing to Bi 6s and O 2pπ hybridization (Figure 8D) [112,113]. These authors also confirmed that this photocatalyst promoted O_2_ evolution under Vis-light irradiation in the presence of a sacrificial agent (Table 1). They also succeeded in increasing the VBM by substituting alkali metal ions with Ag^+^ or Cu^+^ at the near surface of bulky materials, such as NaTaO_3_ (Table 1) [114,115]. Furthermore, there have been several reports of Vis-light-driven water-splitting photocatalysts created by replacing alkali metal ions in the interlayer of layered oxide photocatalysts with Ag^+^ or Cu^+^ (Table 1) [116,117,118].

#### 2.1.2. Formation of Impurity Levels by Doping

**Figure 9:**
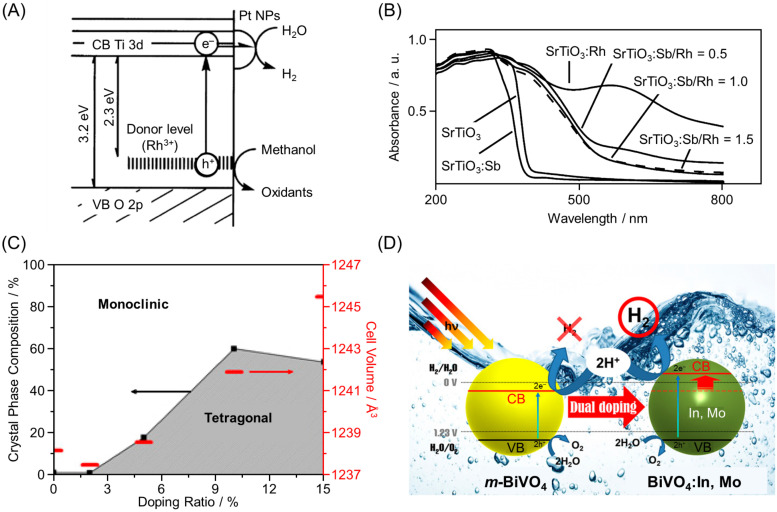
(**A**) Band structure of SrTiO_3_:Rh after photoreduction. (**B**) Diffuse reflection spectra of the six photocatalysts. For example, “SrTiO_3_:Sb/Rh = 0.5” indicates “SrTiO_3_:Sb,Rh in which Sb/Rh = 0.5”. (**C**) Crystal phase diagram with calculated unit cell volume for different doping ratios (atom ratios of In and Mo) of *m*-BiVO_4_. (**D**) Effect of dual doping by In and Mo on the activity of BiVO_4_. Reproduced with permission from references [119,125,128]. Copyright 2004 American Chemical Society, 2013 American Chemical Society, and 2015 National Academy of Sciences.

Metal oxide photocatalysts become Vis-light responsive by forming donor levels (impurity levels) when 0.1% to several percent of the metal ions are substituted with other transition metal ions (such as chromium ion (Cr^3+^), rhodium ion (Rh^3+^), and iridium ion (Ir^3+^)) while maintaining the lattice structure, known as doping. Using such a method, Kudo and co-workers succeeded in developing a Rh-doped strontium titanium oxide (SrTiO_3_:Rh) photocatalyst in 2004 (Figure 7) [119]. For SrTiO_3_:Rh, a donor level (impurity level) was formed at the negative side of VBM (namely, in the forbidden band) due to doping of some Ti^4+^ sites with Rh^3+^ (Figure 9A). For SrTiO_3_:Rh, optical absorption occurred at both 580 and 420 nm immediately after preparation (Figure 9B) [119]. These absorptions were attributed to impurity levels based on Rh^4+^ and Rh^3+^, respectively. However, the acceptor level (impurity level) formed by Rh^4+^ became an exciton recombination center [120]. Therefore, to proceed with the water-splitting reaction efficiently, Rh^4+^ needs to be photoreduced to Rh^3+^ by light irradiation [121]. Furthermore, the formation of such acceptor levels can be avoided by co-doping other metal cations to maintain the charge balance [122,123,124]. For example, Onishi and co-workers succeeded in suppressing the formation of Rh^4+^ by co-doping some Ti^4+^ sites with two types of ion, Sb^5+^ and Rh^3+^ (Figure 9B) [125]. In contrast, Domen and co-workers succeeded in reducing the ratio of Rh^4+^ and increasing the ratio of Rh^3+^ by doping some Ti^4+^ sites with Rh^3+^ and some Sr^2+^ sites with lanthanum ion (La^3+^) (co-doping) [126].

Doping is also effective at shifting the positions of the CB and VB [127]. In 2015, Lee and co-workers simultaneously substituted Bi^3+^ and V^5+^ in *m*-BiVO_4_ with In^3+^ and molybdenum ion (Mo^6+^), respectively, to create BiVO_4_:In,Mo. In this BiVO_4_:In, Mo photocatalyst, BiVO_4_ was a mixture of *m*-BiVO_4_ and tetragonal BiVO_4_ (*t*-BiVO_4_), which induced an increase in the compressive lattice strain (Figure 9C). This caused an increase in the CB and allowed BiVO_4_:In,Mo to promote the HER (Figure 9D) [128].

#### 2.1.3. Narrowing of BG by Solid-Solution Formation

**Figure 10:**
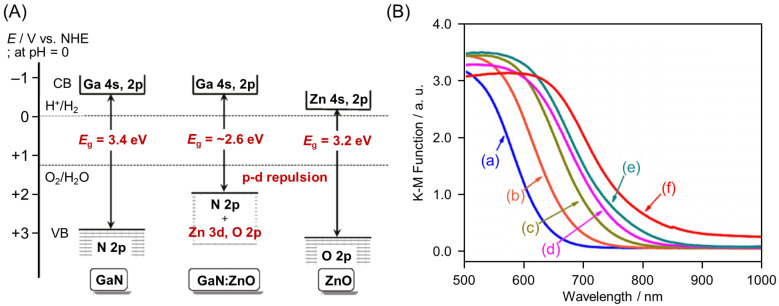
(**A**) Schematic illustration of band structures of GaN, ZnO, and their solid solution. (**B**) Diffuse reflectance spectra for LTCS_1−*x*_Se*_x_*O solid solutions as a function of Se content. Se contents: (a) 0.0, (b) 0.2, (c) 0.4, (d) 0.6, (e) 0.8, and (f) 1.0. Reproduced with permission from references [132,137]. Copyright 2010 American Chemical Society and 2017 Wiley-VCH.

Semiconductor materials with the same crystal structure can easily form a solid solution [136]. The BG and energy levels of the solid solution continuously change depending on the ratio of the two types of semiconductor material [129,130,131,132,133]. Domen and co-workers succeeded in creating GaN:ZnO, which provided the first example of efficient and stable Vis-light-driven OWSR (Figure 10A) by solid-solution formation from GaN (3.4 eV) and ZnO (3.2 eV), which are UV-light-driven photocatalysts [137]. In this solid solution, both Zn 3d and N 2p electrons were present in the VBM, and p–d repulsion occurred between them [137,138], resulting in a narrower BG for this solid solution (~2.6 eV) compared with those of GaN (3.4 eV) and ZnO (3.2 eV) (Figure 10A). In 2017, Domen and co-workers also succeeded in creating La_5_Ti_2_Cu(S_1−*x*_Se*_x_*)_5_O_7_ (LTCS_1−*x*_Se*_x_*), which is a solid solution of La_5_Ti_2_CuS_5_O_7_ (~1.9 eV) and La_5_Ti_2_CuSe_5_O_7_ (1.5 eV) (Figure 7 and Table 1) [132]. The absorption edge of this solid solution shifted monotonically toward longer wavelengths with increasing Se content and, at *x* = 1, the solid solution absorbed light up to 820 nm (Figure 10B). At *x* = 0.2, HER activity was observed under Vis-light irradiation of up to 720 nm (Figure 10B).

#### 2.1.4. Shift of Band Structure by Reducing Particle Size

**Figure 11:**
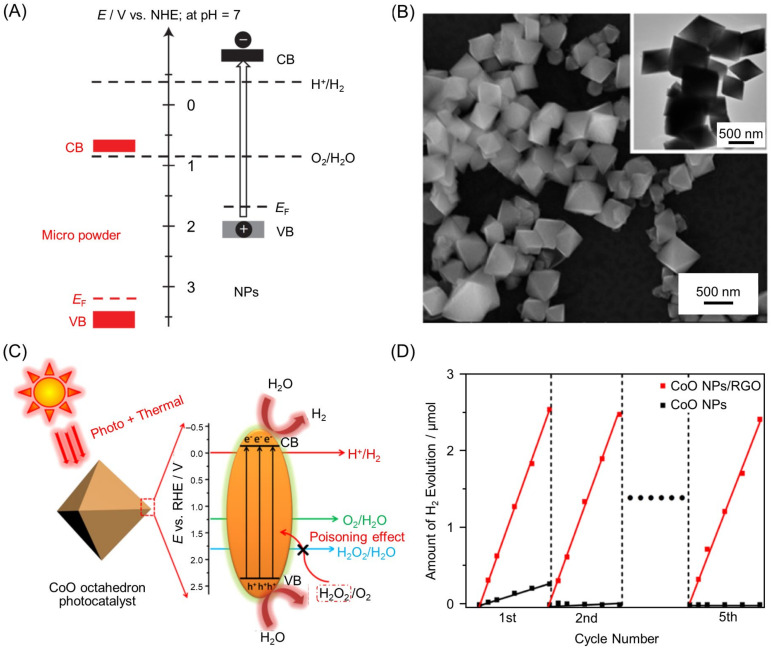
(**A**) Band positions of CoO micropowders and NPs. (**B**) Scanning electron microscopy (SEM) image of CoO octahedra (inset, transmission electron microscope (TEM) image). (**C**) Mechanism of overall water splitting over CoO octahedron photocatalyst. (**D**) Cycle stability of H_2_ evolution from pure water under Vis-light irradiation (λ > 400 nm) using CoO/reduced graphene–oxide (RGO) composite and CoO octahedra (each cycle is 24 h). Reproduced with permission from references [134,135]. Copyright 2014 Springer Nature Limited and 2017 American Chemical Society.

In 2014, Bao and co-workers showed that reducing the size of cobalt (II) oxide (CoO) NPs to ~10 nm shifted their band to a more suitable position for water splitting (Figure 11A). The CoO NPs with a particle size of ~10 nm had a BG (2.6 eV) capable of absorbing Vis light (Figure 7 and Table 1) and induced overall water splitting with a very high efficiency of STH = 5% [134]. Unfortunately, CoO NPs have low stability, like most other Vis-light-driven photocatalysts, and the reaction was deactivated in about 1 h. To address this problem, in 2017, Mao, Liu, Kang, and co-workers created a CoO single crystal with a submicrometer-sized octahedral structure and the active (111) face exposed (Figure 11B) [135]. In this photocatalyst, exposure of the (111) surface suppressed hydrogen peroxide (H_2_O_2_) poisoning, which led to a decrease in photocatalytic activity (Figure 11C). However, even CoO single crystals obtained in this manner did not show sufficient stability, with thermal oxidation of CoO to Co_3_O_4_ observed. Therefore, these authors combined CoO single crystals with graphene, a thermal conductor, to create a highly active and stable Vis-light-driven water-splitting photocatalyst (Figure 11D). Furthermore, in 2017, Kang, Liu, Huang, and co-workers succeeded in improving stability during the degradation reaction of antimicrobial agents by forming a heterojunction of CoO NPs, a p-type semiconductor photocatalyst, and graphitic carbon nitride (g-C_3_N_4_), an n-type semiconductor photocatalyst, to promote charge separation [139]. Lu, Shi, and co-workers have shown that such high stability due to heterojunctions can also be induced using BiVO_4_, an n-type semiconductor photocatalyst [140]. Unfortunately, there have been no reports on the water-splitting activity of such heterojunction photocatalysts using CoO NPs and n-type semiconductor photocatalysts, but water splitting is expected to be studied on such heterojunction photocatalysts in the future.

### 2.2. Use of Other Semiconductor Materials

In recent years, several studies have been reported on the fabrication of Vis-light-driven water-splitting photocatalysts based on semiconductors other than metal oxides. In this section, we describe some representative photocatalysts reported in such studies. The BG energy, type, and loading amount of appropriate cocatalysts, possible reactions (OWSR, HER, or OER; Figure 4), activity, and references for semiconductor photocatalysts described in this section are summarized in Figure 7 and Table 2 [51,141,142,143,144,145,146,147,148,149,150,151,152,153,154].

#### 2.2.1. Connection of Indium Gallium Nitride with Different BGs

**Figure 12:**
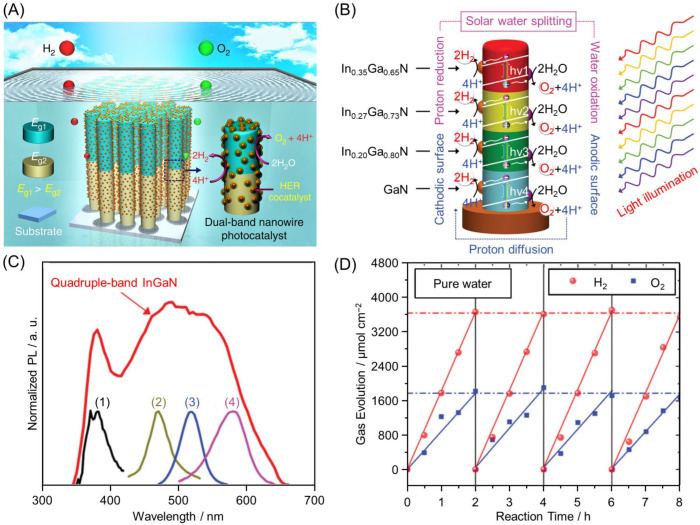
(**A**) Schematic illustration of wafer-level unassisted photocatalytic overall water splitting on dual-band nanowire arrays, which are vertically aligned on a planar substrate and decorated with cocatalysts for HER. Both OER and HER occur on the radial non-polar surfaces of each layer. (**B**) Schematic illustration of the overall water-splitting process occurring on the quadruple-band nanowires under light irradiation. (**C**) Room-temperature photoluminescence (PL) spectrum of the quadruple-band InGaN nanowires, together with those of single-band nanowires including (1) p-type GaN, (2) p-In_0.20_Ga_0.80_N, (3) p-In_0.27_Ga_0.73_N, and (4) p-In_0.35_Ga_0.65_N. (**D**) Cycle stability of H_2_ and O_2_ evolution from pure water using quadruple-band InGaN nanowire arrays with a 300 W xenon (Xe) lamp equipped with an AM1.5G filter (each cycle is 2 h). Reproduced with permission from references [141,155]. Copyright 2018 Springer Nature Limited and 2019 The Royal Society of Chemistry.

For indium gallium nitride (InGaN), the BG varies continuously from the UV to near-infrared regions depending on the composition of In and Ga. In 2018, Vayssieres, Mi, and co-workers developed a photocatalyst with a dual-band structure (Figure 12A) by combining this material with GaN (3.4 eV) [155]. In 2019, these authors also succeeded in creating a nanowire photocatalyst with a quadruple-band structure (Figure 12B) composed of In_0.35_Ga_0.65_N (2.1 eV), In_0.27_Ga_0.73_N (2.4 eV), In_0.20_Ga_0.80_N (2.6 eV), and GaN (3.4 eV) [141]. The multiband InGaN nanowires were directly grown on a non-planar silicon wafer, had a large surface area, and absorbed light with high efficiency. Furthermore, the combination of four types of photocatalysts with different BGs allowed this photocatalyst to absorb sunlight across almost the entire Vis-light spectrum (Figure 12C). The obtained photocatalyst maintained its water-splitting activity for a long time under Vis-light irradiation (Figure 12D).

#### 2.2.2. Use of Metal-Free Semiconductors

##### g-C_3_N_4_

**Figure 13:**
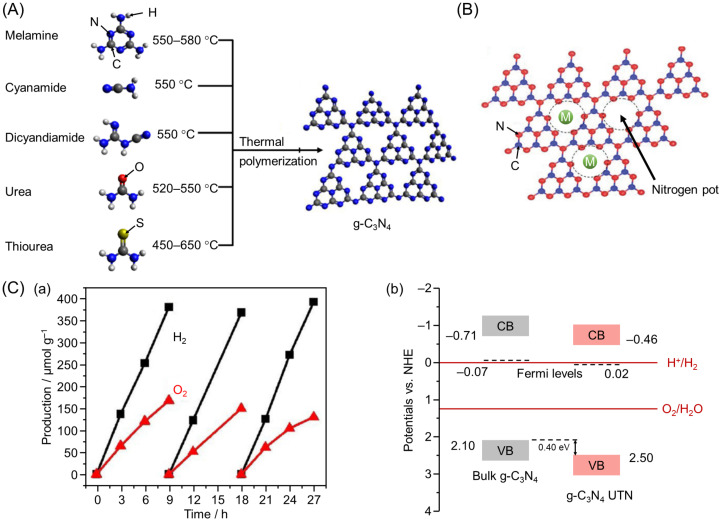
(**A**) Schematic illustration of the synthesis process of g-C_3_N_4_ by thermal polymerization of different precursors, such as melamine, cyanamide, dicyanamide, urea, and thiourea. Black, blue, white, red, and yellow balls denote C, N, H, O, and S atoms, respectively. (**B**) Schematic diagram of a perfect g-C_3_N_4_ sheet constructed from tri-*s*-triazine units. Red and blue balls denote N and C atoms, respectively. ○ indicates the nitrogen pot filled with six nitrogen lone-pair electrons, which are potentially ideal sites for metal (M) inclusion. (**C**) (**a**) The photocatalytic overall water splitting of g-C_3_N_4_ ultra-thin nanosheets with ∼1.4 wt% of Pt atoms as cocatalyst from pure water under Vis-light irradiation (λ > 400 nm). (**b**) The band edge positions of bulk g-C_3_N_4_ and g-C_3_N_4_ ultra-thin nanosheets. Reproduced with permission from references [152,156,171]. Copyright 2016 American Chemical Society, 2014 The Royal Society of Chemistry, and 2021 Elsevier.

As mentioned above, the constituent elements of Vis-light-driven water-splitting photocatalysts reported to date often include rare metals (such as Ti, V, Cr, manganese (Mn), Co, Ga, Se, Sr, Zr, Nb, Mo, Sb, Ta, and Bi) and rare earths (such as La) [94,95,96,97,98,100,101,102,103,108,109,110,112,114,116,117,118,119,120,128,129,131,132,134,141]. Despite their limited reserves, these metals are used in various industries, such as semiconductors and automobiles. Therefore, if Vis-light-driven water-splitting photocatalysts were implemented in society, the cost of these raw materials would soar, and the price of H_2_ might continue to increase. Accordingly, the development of Vis-light-driven water-splitting photocatalysts that are free of rare metals and rare earths is also currently in progress.

g-C_3_N_4_ is a metal-free organic semiconductor photocatalyst that can be readily synthesized by thermal polymerization of N-containing precursors, such as urea, melamine, and cyanamide (Figure 13A) [156,157,158,159]. g-C_3_N_4_ can also be synthesized at low cost because these precursors are earth-abundant. Furthermore, for g-C_3_N_4_ (BG = 2.7–2.9 eV; Figure 7 and Table 2), the CBM is composed of C p_z_ orbitals, and the VBM is composed of N p_z_ orbitals, with band positions suitable for water splitting [160]. Accordingly, g-C_3_N_4_ has attracted significant attention as a next-generation Vis-light-driven water-splitting photocatalyst [161,162,163,164,165,166,167,168,169,170,171,172,173].

The electronic structure of g-C_3_N_4_ can also be changed by the substitution or introduction of different atoms. Many examples of modifying the electronic structure of g-C_3_N_4_ by substitution with O, C, P, S, boron (B), I, and F have been reported (Figure 7 and Table 2) [142,143,144,145,146,147,148,170]. For example, substituting some of the N in g-C_3_N_4_ with S shifts both the VBM and CBM to the negative side, resulting in improved HER activity of the photocatalyst [144]. In addition to these substitutions, incorporating metal cations, such as ferric ion (Fe^3+^), Cu^2+^, Zn^2+^, and nickel ion (Ni^3+^), into the nitrogen pot of g-C_3_N_4_ (Figure 13B) has been shown to change the optical and electronic properties (such as reduced BG, accelerated charge transfer, and extended charge carrier lifetime) [149,150,151,171].

In addition, several studies have shown that OWSR can be achieved by changing the morphology of g-C_3_N_4_ [172,173]. For example, in 2021 Bao, Pu, and Wang successfully exfoliated g-C_3_N_4_ by irradiating bulk g-C_3_N_4_ with a femtosecond pulsed laser and thereby synthesized g-C_3_N_4_ ultra-thin nanosheets (UTN) [152]. The deposition of platinum (Pt) single atoms on g-C_3_N_4_ UTN resulted in OWSR with a H_2_ production rate of 42.6 µmol g^−1^ h^−1^ and an O_2_ production rate of 18.7 µmol g^−1^ h^−1^ (Figure 13C(a)). They attributed this phenomenon to the formation of C≡N defects caused by laser stripping, which induced the two following things: (1) aiding the loading of Pt single atoms and thereby increasing the number of active sites, and (2) downshifting the CBM and VBM to promote the OER (Figure 13C(b)).

##### Connection of Covalent Organic Frameworks with Metal–Organic Frameworks or Covalent Organic Frameworks

Covalent organic frameworks (COFs) and metal–organic frameworks (MOFs) are crystalline and porous materials that consist of pure organic molecules and metals bonded by covalent bonds or coordination bonds. They are used in various fields, such as gas storage, catalysis, and sensing. COFs have long-range ordered structure, excellent surface area, and adjustable BG and have recently attracted much attention as water-splitting photocatalysts [174]. Most two-dimensional COFs reported to date form layered structures through π–π stacking, which allows them to transport charge carriers not only within but also between covalent sheets [175,176,177]. Furthermore, most COFs (especially Schiff-based COFs) have colors ranging from orange to dark red and provide excellent light harvesting in the Vis-light range. However, COFs not only exhibit low hydrophilicity and stability but also easily cause the recombination of photoexcited carriers. Therefore, the water-splitting activity of COFs has generally been lower than those of metal oxide (sulfide) Vis-light-driven water-splitting photocatalysts.

**Figure 14:**
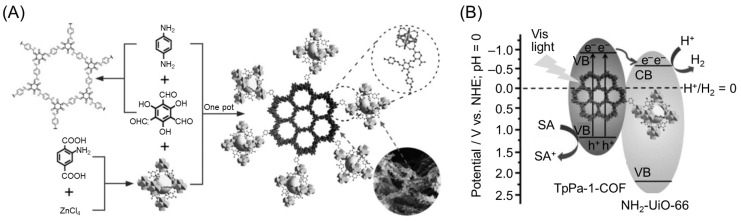
(**A**) Schematic illustration of the synthesis of the hybrid material composed of TpPa-1-COF (COF; upper compounds) and NH_2_-UiO-66 (MOF; lower compounds). (**B**) Schematic of mechanism of HER over the hybrid material (TpPa-1-COF:NH_2_-UiO-66 = 4:6 (optimal ratio)). SA represents the sodium ascorbate. Reproduced with permission from reference [153]. Copyright 2018 Wiley-VCH.

In 2018, Lan and co-workers reported the development of a hybrid photocatalyst of a COF (TpPa-1-COF) and a MOF (NH_2_-UiO-66) [153]. The hybrid photocatalyst had a band structure suitable for charge separation, and the COF and MOF were strongly connected by covalent bonds (Figure 14A). In these hybrid photocatalysts, the transfer of excited electrons occurred efficiently (Figure 14B), resulting in high HER activity (Table 2).

**Figure 15:**
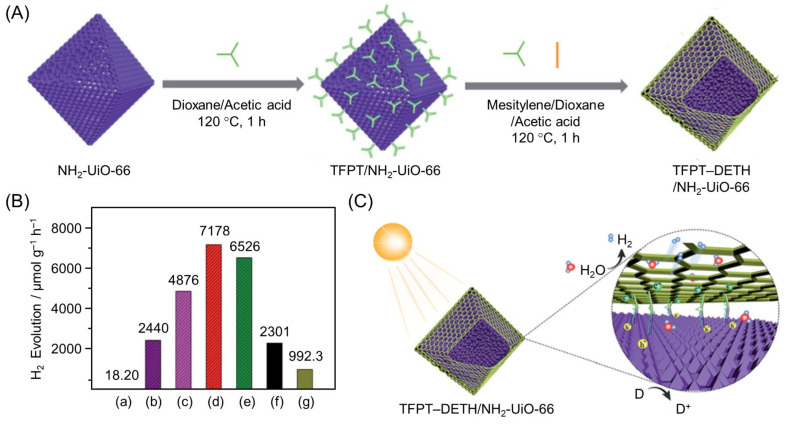
(**A**) Schematic illustration of the synthetic process of TFPT–DETH/NH_2_-UiO-66 core–shell hetero-frameworks. (**B**) Corresponding HER rates of photocatalysts using phosphate-buffered saline buffer solution (pH = 7.4) containing 100 mg of sodium ascorbate as a sacrificial electron donor, and Pt NPs (7.5 wt%) as a cocatalyst, under Vis-light irradiation (λ ≥ 420 nm, Xe lamp): (a) NH_2_-UiO-66, (b–e) TFPT–DETH/NH_2_-UiO-66 (*n*) samples (*n* = 1, 2, 4, or 6), (f) TFPT–DETH, and (g) physical mixture of TFPT–DETH and NH_2_-UiO-66 (TFPT–DETH:NH_2_-UiO-66 = 1:1). (**C**) Schematic illustration of photocatalytic H_2_ evolution over the TFPT–DETH/NH_2_-UiO-66 (*n*) hetero-framework under Vis-light irradiation. In the figure, D denotes sodium ascorbate. Reproduced with permission from reference [154]. Copyright 2019 The Royal Society of Chemistry.

Other groups have also been working on the development of such hybrid photocatalysts. For example, using a MOF (NH_2_-UiO-66) and a COF (1,3,5-tris-(4-formyl-phenyl) triazine (TFPT)–2,5-diethoxybenzene-1,4-dicarbohydrazide (DETH)), Jiang and co-workers successfully created octahedral core–shell hetero-framework photocatalysts (TFPT–DETH/NH_2_-UiO-66) [154]. These photocatalysts were formed by epitaxial growth of a TFPT–DETH shell on a NH_2_-UiO-66 core (Figure 15A). The authors obtained a series of TFPT–DETH/NH_2_-UiO-66 samples (TFPT–DETH/NH_2_-UiO-66 (*n*) (*n* = 1, 2, 4, or 6)) with different shell thicknesses by varying the amounts of TFPT and DETH. As shown in Figure 15B, the HER rate increased with increasing TFPT shell thickness, reaching a maximum at DETH/NH_2_-UiO-66 (4), and then steadily decreased. These phenomena were due to some of the bulk material, including the MOF core, not being able to absorb sufficient light when the COF shell was too thick, which reduced the amount of photogenerated excitons. Furthermore, for organic semiconductors, a thicker shell results in a faster exciton recombination rate inside the photocatalyst owing to the shorter diffusion length and lower mobility of excitons. Owing to these factors, the photocatalytic activity of DETH/NH_2_-UiO-66 (*n*) samples gave the highest HER rate when the thickness of the COF shell was at the optimum value (*n* = 4). The DETH/NH_2_-UiO-66 (4) photocatalyst showed excellent HER activity with an apparent quantum yield (AQY) of 1.11% (at 420 nm). The authors suggested that synergistic effects in the hetero-framework were responsible for this high AQY (Figure 15C).

## 3. Control of Cocatalysts

As described in Section 2, the development of Vis-light-driven water-splitting photocatalysts has seen rapid progress in recent years (Figure 7, Table 1 and Table 2). However, water splitting was only achieved using a semiconductor photocatalyst alone in a few reported cases. This is largely due to two factors, as follows: (i) Most of the photoexcited charges recombine in a short time and relax energetically, producing heat (non-radiative deactivation) and luminescence (radiative deactivation); and (ii) the surface of the semiconductor photocatalyst has few active sites for reaction. Therefore, for the photocatalytic reaction to proceed with a high quantum yield (QY), electrons and holes must be spatially separated and transferred to active sites on the surface before recombination.

**Figure 16:**
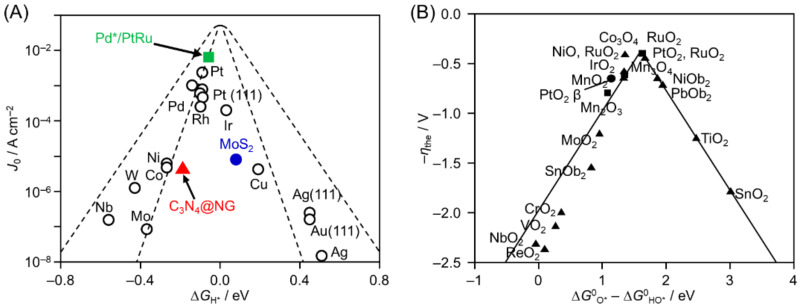
(**A**) The HER *j*_0_ (exchange current density) values as a function of the free energy from the adsorption of H_2_ (∆G_H*_) for the surfaces of various metals, alloys, and nonmetallic materials. (**B**) −*η*_the_ (negative values of theoretical overpotential) values for the OER as a function of (∆G^0^_O*_−∆G^0^_HO*_). Reproduced with permission from references [178,217]. Copyright 2014 Springer Nature Limited and 2014 Wiley-VCH.

Cocatalysts promote the transfer of excitons generated in the photocatalyst and act as active sites to lower the activation energy of the HER and OER. When selecting a suitable HER cocatalyst, the volcano plot (Figure 16A) obtained from the results of Langmuir-type adsorption of H_2_ is often used [178,179,180]. This volcano plot shows that the catalyst surface with the optimal binding energy to reactants and products has the highest catalytic activity. Therefore, noble metals, such as Pt [181,182,183,184] and Rh [104,185], for which the H_2_ adsorption free energy (∆G_H*_) is near 0, are often used as elements in HER cocatalysts. Recently, HER cocatalysts without precious metal elements (such as MoS_2_ [186,187,188,189], NiS [41,190,191,192,193,194,195], CoMoS [196,197,198,199], Ni_2_P [200,201,202,203,204], and FeP [205,206,207]) have also been developed. Meanwhile, metal oxides (sulfides), such as CoO*_x_* [208,209,210], RuO_2_ [211,212,213], IrO_2_ [120,214], and PdS [215,216], are often used as OER cocatalysts, based on the volcano plot shown in Figure 16B [217]. 

**Figure 17:**
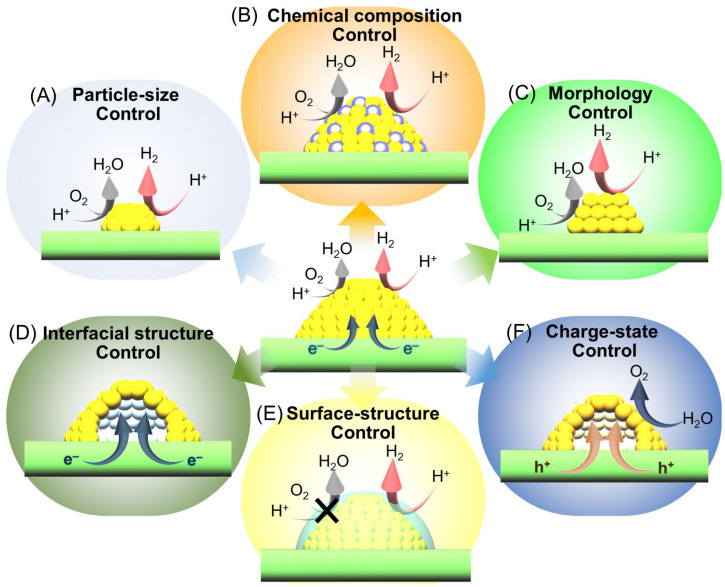
Schematic of photocatalyst functionalization by controlling cocatalyst properties: (**A**) particle size (Section 3.1), (**B**) chemical composition (Section 3.2), (**C**) morphology (Section 3.3), (**D**) interfacial structure (Section 3.4), (**E**) surface structure (Section 3.5), and (**F**) charge state (Section 3.6).

Thus, each reaction consists of the adsorption of reactants on the cocatalyst surface, reaction on the cocatalyst surface, and desorption from the cocatalyst surface, and these processes are largely related to the adsorption and desorption energy at the cocatalyst surface. Therefore, these properties need to be considered when improving the functionality of the photocatalyst by modifying the cocatalyst. This section discusses six approaches to improving such cocatalysts, as follows: (i) particle-size control (Section 3.1; Figure 17A) [73,74,75,77,218]; (ii) chemical composition control (Section 3.2; Figure 17B) [76,219,220]; (iii) morphology control (Section 3.3; Figure 17C) [221,222,223,224]; (iv) interface structure control (Section 3.4; Figure 17D) [225]; (v) surface-structure control (Section 3.5; Figure 17E) [77,226,227,228,229,230,231,232,233,234,235,236,237,238,239,240,241]; and (vi) charge-state control (Section 3.6; Figure 17F) [242,243]. For the semiconductor photocatalysts described in this section, the appropriate cocatalysts, possible reactions (OWSR, HER, or OER; Figure 4), activities, and references are summarized in Table 3 [74,75,76,77,218,219,220,221,222,223,224,225,234,241,242,243].

### 3.1. Particle Size Control

**Figure 18:**
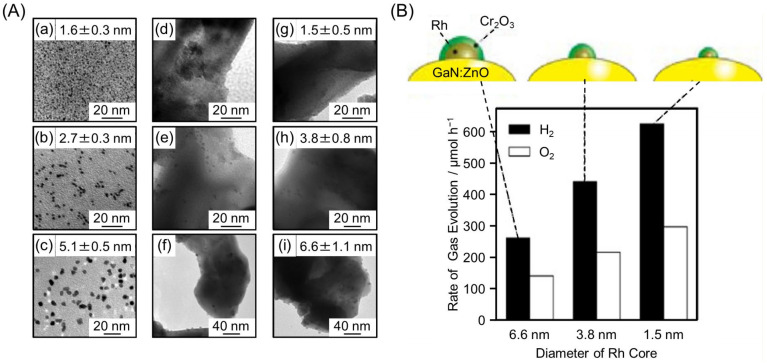
(**A**) TEM images of three types of Rh NPs: (**a**–**c**) as-synthesized, (**d**–**f**) adsorbed on GaN:ZnO, and (**g**–**i**) loaded on GaN:ZnO after calcination. (**B**) Initial rates of H_2_ and O_2_ evolution over GaN:ZnO loaded with different-sized Cr_2_O_3_/Rh (core–shell) NPs. Reaction conditions: catalyst, 0.15 g; H_2_SO_4_ aq. (pH 4.5), 400 mL; light source, high-pressure Hg lamp (450 W) through NaNO_2_ aq. filter to cut UV light; reaction vessel, Pyrex inner-irradiation type. Reproduced with permission from reference [218]. Copyright 2013 American Chemical Society.

**Figure 19:**
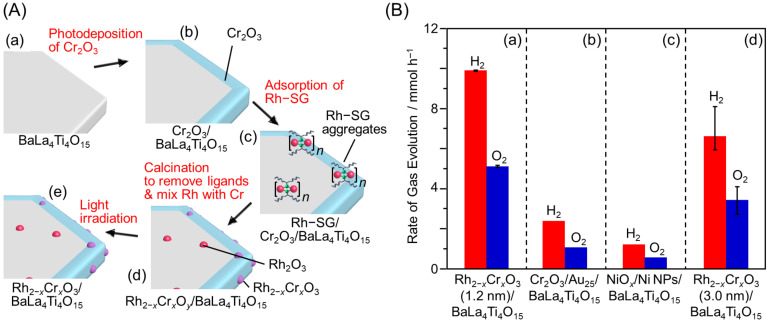
(**A**) Schematic of experimental procedure for the formation of BaLa_4_Ti_4_O_15_ loaded with small Rh_2−_*_x_*Cr*_x_*O_3_ NCs (1.2 nm) cocatalysts (Rh_2−_*_x_*Cr*_x_*O_3_ (1.2 nm)/BaLa_4_Ti_4_O_15_). (**a**) BaLa_4_Ti_4_O_15_, (**b**) Cr_2_O_3_/BaLa_4_Ti_4_O_15_, (**c**) Rh–SG/Cr_2_O_3_/BaLa_4_Ti_4_O_15_, (**d**) Rh_2−_*_x_*Cr*_x_*O*_y_* (1.2 nm)/BaLa_4_Ti_4_O_15_, and (**e**) Rh_2−_*_x_*Cr*_x_*O_3_ (1.2 nm)/BaLa_4_Ti_4_O_15_. Rh_2−_*_x_*Cr*_x_*O*_y_* represents Rh_2−_*_x_*Cr*_x_*O_3_ containing highly oxidized Cr (> 3+). (**B**) Comparison of H_2_ and O_2_ evolution rates from pure water using a high-pressure Hg lamp (400 W) over different photocatalysts: (**a**) Rh_2−_*_x_*Cr*_x_*O_3_ (1.2 nm)/BaLa_4_Ti_4_O_15_ (0.09 wt% Rh and 0.10 wt% Cr), (**b**) Cr_2_O_3_/Au_25_/BaLa_4_Ti_4_O_15_ (0.10 wt% Au and 0.50 wt% Cr), (**c**) NiO*_x_*/Ni NPs/BaLa_4_Ti_4_O_15_ (0.50 wt% Ni), and (**d**) Rh_2−_*_x_*Cr*_x_*O_3_ (3.0 nm)/BaLa_4_Ti_4_O_15_ (0.10 wt% Rh and 0.15 wt% Cr). In this study, NiO*_x_*/Ni NPs/BaLa_4_Ti_4_O_15_ and Rh_2−_*_x_*Cr*_x_*O_3_ (3.0 nm)/BaLa_4_Ti_4_O_15_ were prepared using the impregnation method. Reproduced with permission from reference [77]. Copyright 2020 Wiley-VCH.

The size of cocatalyst NPs has a significant effect on the photocatalytic activity. For example, Teranishi and co-workers synthesized monodisperse Rh NPs of different sizes (1.6 ± 0.5, 2.7 ± 0.3, or 5.1 ± 0.5 nm) using a polyol synthesis method, and then this HER cocatalyst was loaded on GaN:ZnO (Rh NPs/GaN:ZnO) by removing the ligands via calcination [218]. Transmission electron microscopy (TEM) images (Figure 18A) showed that the Rh NPs cocatalyst was slightly aggregated on GaN:ZnO but still highly monodisperse (1.5 ± 0.3, 3.8 ± 0.8, or 6.6 ± 1.1 nm). A Cr_2_O_3_ shell (see Section 3.5.1) was then formed on the Rh NPs (Cr_2_O_3_/Rh NPs/GaN:ZnO), which acted as a reverse-reaction suppressor layer. The water-splitting activity of Cr_2_O_3_/Rh NPs/GaN:ZnO was enhanced with a reduction in size of the Rh NPs cocatalyst (Figure 18B and Table 3). These size effects were attributed to an increase in the proportion of surface atoms (HER active sites) in the Rh NPs cocatalyst, and enhanced charge separation as the Rh NPs cocatalyst decreased in size. Such an enhancement of the water-splitting activity caused by a reduction in size of the cocatalyst has also been observed in our study on gold (Au)-NCs or Rh_2−*x*_Cr*_x_*O_3_-NCs-loaded BaLa_4_Ti_4_O_15_ photocatalysts (UV-light-driven photocatalysts; Figure 19) [73,74,75,77].

### 3.2. Chemical Composition Control

**Figure 20:**
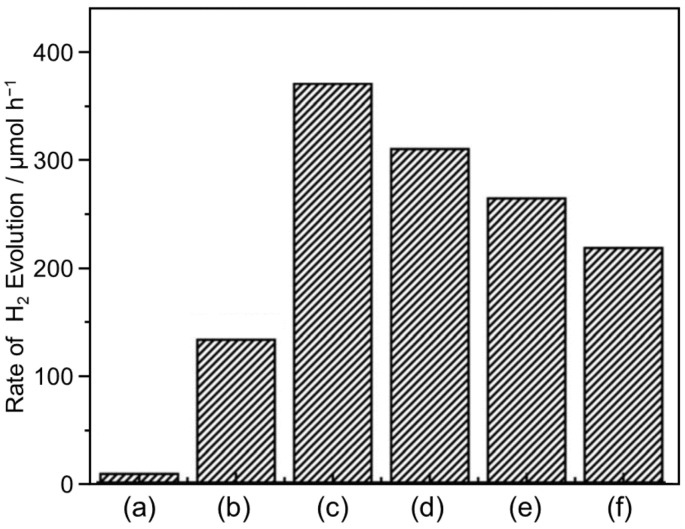
Photocatalytic H_2_ evolution from 50 vol% aqueous methanol solution under high-pressure Hg lamp irradiation over (a) bare SrTiO_3_ and (b–f) Cu_1−*x*_Pt*_x_*/SrTiO_3_ (*x* = (b) 0, (c) 0.05, (d) 0.1, (e) 0.9, or (f) 1.0) NPs. Reproduced with permission from reference [219]. Copyright 2015 The Royal Society of Chemistry.

Alloying enables the creation of materials with physical properties and functions different from those of single metals. In 2015, Qin, Kang, and co-workers showed that the HER activity of SrTiO_3_ was significantly enhanced when Cu–Pt alloy NPs were used as the cocatalyst compared with using Cu NPs or Pt NPs as the cocatalyst [219]. Specifically, Cu_0.95_Pt_0.05_ NPs/SrTiO_3_ showed an HER rate about 2.79, 1.76 times higher than those of Cu NPs/SrTiO_3_ and Pt NPs/SrTiO_3_, respectively (Figure 20 and Table 3). The authors’ interpretation of these results was that using Cu–Pt alloy NPs as the cocatalyst led to high HER rates because excited electrons were efficiently transferred to the cocatalyst, which suppressed charge recombination.

**Figure 21:**
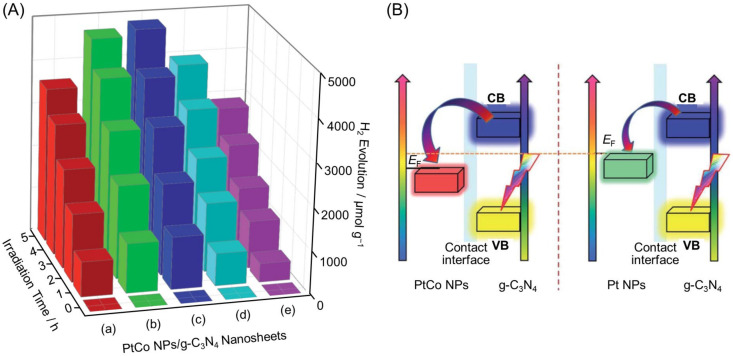
(**A**) Photocatalytic H_2_ evolution from aqueous solution containing 10 vol% of triethanolamine (sacrificial reagent) under Vis-light irradiation (≥400 nm) over Pt_1__−_*_x_*Co*_x_* NPs/g-C_3_N_4_ nanosheets with different Pt/Co percentages at the same loading of 1.0 wt%; *x* = (a) 0, (b) 0.25, (c) 0.5, (d) 0.75, and (e) 0.86. (**B**) Schematic illustration of photoexcited electron injection from CB of g-C_3_N_4_ to the PtCo NPs and Pt NPs. Reproduced with permission from reference [220]. Copyright 2015 The Royal Society of Chemistry.

In 2015, Ge and co-workers reported using an alloy consisting of Pt and Co as a cocatalyst [220]. In this study, Pt_0.5_Co_0.5_ NPs/g-C_3_N_4_ nanosheets were found to have HER activity 1.34 times higher than that of Pt NPs/g-C_3_N_4_ nanosheets with the same Pt loading (Figure 21A and Table 3). Alloying with Co was interpreted to increase the driving force for photoexcited electron transfer from the CB of g-C_3_N_4_ to the cocatalyst, resulting in enhanced HER activity (Figure 21B). However, when the Co content exceeded the Pt content, the HER activity decreased (Figure 21A). This was attributed to the reduction in HER reaction sites on the cocatalyst surface when the Co content became too large.

**Figure 22:**
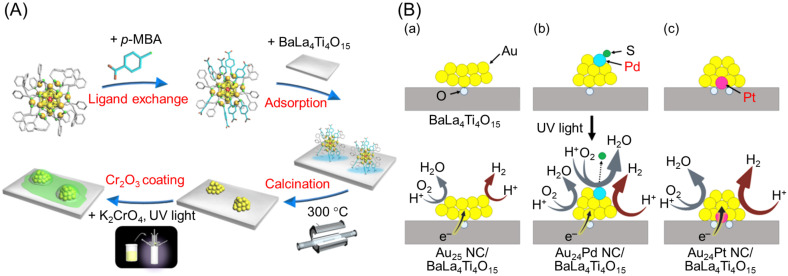
(**A**) Schematic illustration of the experimental procedure for the preparation of Au_24_M NC/BaLa_4_Ti_4_O_15_ (M = Au, Pd, or Pt). *p*-MBA; 4-mercaptobenzoic acid. (**B**) Proposed structures of Au_24_M NC/BaLa_4_Ti_4_O_15_, where M is (**a**) Au, (**b**) Pd, and (**c**) Pt before (above) and during (below) the water-splitting reaction. Reproduced with permission from reference [76]. Copyright 2019 American Chemical Society.

As described above, alloying is extremely effective at improving the cocatalyst function. Recently, the synthesis of alloy NCs with precisely controlled chemical compositions by liquid-phase synthesis has become possible [244,245,246,247,248,249,250,251,252,253,254,255,256,257,258,259,260,261,262,263,264,265,266,267,268,269,270,271,272,273,274,275,276,277,278,279,280,281,282,283]. The use of these fine alloy NCs as precursors will allow precise control of the chemical composition of loaded metal NCs (Figure 22), which is expected to provide an improved understanding of the factors that contribute to the enhanced activity induced by alloying [86].

### 3.3. Morphology Control

When metal NPs are used as a cocatalyst, the water-splitting activity changes depending on the exposed crystal plane. This is due to the electronic structure and surface energy of the metal NPs’ surface differing depending on the crystal plane, resulting in different adsorption properties with the substrate. The optimal shape and crystal planes are different depending on the metal species.

**Figure 23:**
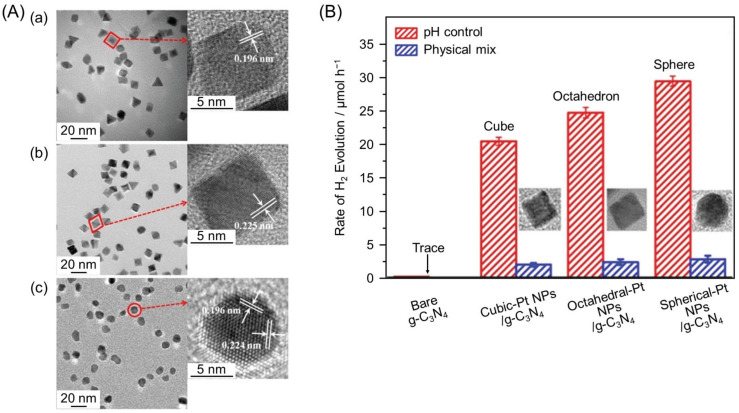
(**A**) TEM images and size distributions of dominant (**a**) cubic-, (**b**) octahedral-, and (**c**) spherical-Pt NPs. Insets show high-resolution (HR)-TEM images of a single Pt nanocrystal for each shape. (**B**) Photocatalytic H_2_ evolution activities of bare g-C_3_N_4_, as-prepared Pt NPs/g-C_3_N_4_ photocatalysts, and physically mixed samples under Vis-light irradiation (λ > 400 nm) within 1 h. Reproduced with permission from reference [221]. Copyright 2016 The Royal Society of Chemistry.

In 2016, Yu and co-workers synthesized Pt NPs with different shapes (cubic, octahedral, or spherical; ~10 nm; Figure 23A) and successfully loaded them on g-C_3_N_4_ [221]. Studies on the as-obtained Pt NPs/g-C_3_N_4_ photocatalysts showed that their HER activity increased in the order of cubic-Pt NPs/g-C_3_N_4_ < octahedral-Pt NPs/g-C_3_N_4_ < spherical-Pt NPs/g-C_3_N_4_ (Figure 23B and Table 3). The cubic-Pt NPs consist of six (100) planes and do not have many active sites, consisting of sharp edges and corners, which are necessary for HER evolution. In contrast, octahedral-Pt NPs are composed of eight (111) planes and have more active sites, consisting of sharp edges and corners, than cubic-Pt NPs. Meanwhile, spherical-Pt NPs are composed of a large number of (100) and (111) planes, and these structures contain many active sites consisting of sharp edges and corners. These factors were interpreted to be related to the HER activity of Pt NPs/g-C_3_N_4_ with Pt NPs cocatalysts of different geometries, in the order of cubic < octahedral < spherical.

**Figure 24:**
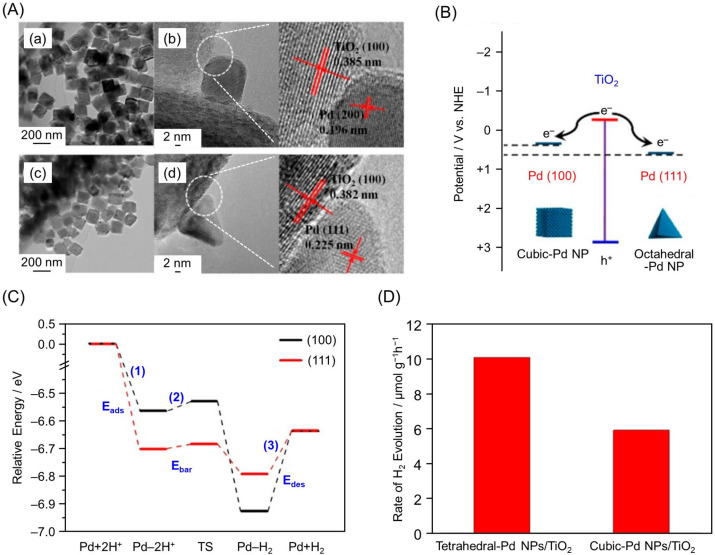
(**A**) TEM images of (**a**) cubic-Pd NPs/TiO_2_ and (**c**) tetrahedral-Pd NPs/TiO_2_ samples, and HR-TEM images of (**b**) cubic-Pd NPs/TiO_2_ and (**d**) tetrahedral-Pd NPs/TiO_2_ samples. (**B**) Schematic illustration of photogenerated electron transfer from the CB of TiO_2_ to the Pd (100) and Pd (111) surface. (**C**) Energy variation in the H_2_ evolution process on Pd (100) and Pd (111) facets. The total energy of the initial Pd surface and two isolated H atoms is set to zero. Parenthesized numbers indicate the three reaction steps. TS represents the transition state. *E*_ads_, *E*_bar_, and *E*_des_ are the adsorption energy of H atoms, energy barrier, and desorption energy of the H_2_ molecule, respectively. The calculated *E*_abs_, *E*_bar_, and *E*_des_ of Pd (100) facets are −6.57, 0.034, and 0.29 eV, respectively, and those of Pd (111) facets are −6.77, 0.018 and 0.16 eV, respectively. (**D**) Photocatalytic H_2_ evolution activities of tetrahedral-Pd NPs/TiO_2_ and cubic-Pd NPs/TiO_2_ from 10 vol% lactic acid solution under Vis-light irradiation (λ > 420 nm). Reproduced with permission from reference [222]. Copyright 2018 American Chemical Society.

Similar shape dependence has also been observed for TiO_2_ loaded with Pd NPs. In 2018, Yu and co-workers prepared Pd NPs/TiO_2_ photocatalysts loaded with cubic or tetrahedral Pd nanocrystals (cubic-Pd NPs/TiO_2_ and tetrahedral-Pd NPs/TiO_2_, respectively; Figure 24A) and investigated their HER activity [222]. The results demonstrated that tetrahedral-Pd NPs/TiO_2_ showed HER activity 1.5–2.0 times higher than that of cubic-Pd NPs/TiO_2_ (Table 3). The tetrahedral-Pd NPs consisted of four (111) planes, to which photoexcited electron transfer from the CB of TiO_2_ proceeded efficiently (Figure 24B). On the tetrahedral-Pd NPs surface, adsorption of H, H-to-H_2_ conversion of molecules, and desorption of H_2_ molecules readily occurred (Figure 24C). Furthermore, theoretical calculations indicated that the (111) planes had a large work function, and that not only the edge/corner atoms but also the uncoordinated surface atoms, could be reaction sites. Accordingly, tetrahedral-Pd NPs/TiO_2_ exhibited higher HER activity compared with cubic-Pd NPs/TiO_2_ (Figure 24D).

**Figure 25:**
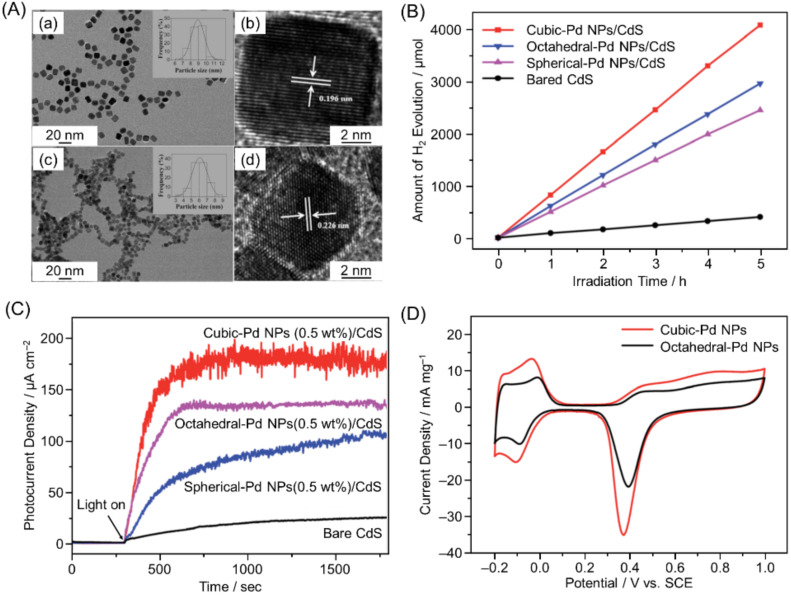
(**A**) TEM images of (**a**) cubic-Pd NPs and (**c**) octahedral-Pd NPs, and HR-TEM images of single (**b**) cubic-Pd NPs and (**d**) octahedral-Pd NPs. Insets in (**a**) and (**c**) are the size distributions of each Pd nanocrystal. (**B**) H_2_ evolution from 0.125 M ammonium sulfite ((NH_4_)_2_SO_3_)•H_2_O aqueous solution under Vis-light irradiation (λ > 420 nm) using cubic-Pd NPs/CdS, octahedral-Pd NPs/CdS, and spherical-Pd NPs/CdS photocatalysts. (**C**) Electron shuttle-mediated photocurrent measurements in suspensions of CdS and three types of Pd NPs-deposited CdS. Experimental conditions: Pd/CdS concentration, 0.5 g L^–1^ of photolyte; aqueous photolyte composition, 0.1 M of sodium nitrate (NaNO_3_)/1.0 mM of FeCl_3_/10 vol% CH_3_OH; light source, 300 W Xe light with cutoff filter (λ > 420 nm); bias potential, 0.6 V (vs. saturated calomel electrode (SCE)). (**D**) Cyclic voltammetry curves of cubic-Pd NPs and octahedral-Pd NPs derived from 0.1 M of HClO_4_ solution at a scan rate of 50 mV s^−1^. Reproduced with permission from reference [223]. Copyright 2015 The Royal Society of Chemistry.

In contrast, in a 2015 study on Pd NPs/cadmium sulfur (CdS), Yao and co-workers reported a different shape dependence than the above two reports [223]. In this study, the authors synthesized cubic-Pd NPs (~8.9 nm) surrounded by six (100) planes and octahedral-Pd NPs (~6.0 nm) surrounded by eight (111) planes (Figure 25A) and loaded them on CdS photocatalysts. From photocatalytic activity measurements, it was found that cubic-Pd NPs/CdS shows higher HER activity compared with octahedral-Pd NPs/CdS (Figure 25B). The photocurrent generation efficiency of cubic-Pd NPs/CdS was higher than that of octahedral-Pd NPs/CdS (Figure 25C), which indicated that electron transfer from the cocatalyst to the reactants was more efficient in the former. Estimation of the electrochemical surface area (ECSA) (Figure 25D) showed that the cubic-Pd NPs/CdS (21.7 m^2^ g^−1^ Pd) had an ECSA 1.49 times higher than that of octahedral-Pd NPs/CdS (14.6 m^2^ g^−1^ Pd), meaning that more proton adsorption/desorption sites were present in the former. These were attributed as the two main factors causing cubic-Pd NPs/CdS to show higher HER activity compared with octahedral-Pd NPs/CdS.

**Figure 26:**
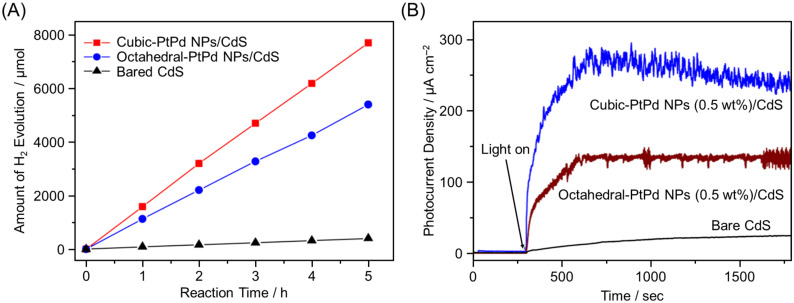
(**A**) Irradiation time course for H_2_ evolution from 1.0 M of aqueous (NH_4_)_2_SO_3_ solution under Vis-light irradiation (λ > 420 nm) using cubic-PtPd NPs/CdS, octahedral-PtPd NPs/CdS, and bare-CdS photocatalysts. (**B**) Electron shuttle-mediated photocurrent measurements in suspensions of CdS, cubic-PtPd NPs/CdS, and octahedral-PtPd NPs/CdS. Conditions: Catalyst concentration, 0.5 g L^−1^ of photolyte; aqueous photolyte composition, 0.1 M of NaNO_3_/1.0 mM of FeCl_3_/10 vol% CH_3_OH; light source, 300 W Xe light with a cutoff filter (λ > 420 nm); bias potential, 0.6 V (vs. SCE)). Reproduced with permission from reference [224]. Copyright 2016 American Chemical Society.

In the above studies, one metal element was used in the cocatalyst. However, in 2016, Yao, Xu, and co-workers reported using two elements in the cocatalyst. The authors found that the HER activity of Pt–Pd alloy NPs/CdS depended on both the shape and composition of the alloy NPs cocatalyst [224]. Regarding this shape dependence, cubic-PtPd NPs/CdS had a much higher HER activity (Figure 26A) and a 3.4 times higher photocatalytic turnover frequency (TOF) compared with octahedral-PtPd NPs/CdS. Electrochemical experiments (Figure 26B) showed that the interfacial electron transfer rate in cubic-PtPd NPs/CdS was higher than that in octahedral-PtPd NPs/CdS. Regarding the composition dependence, H_2_ evolution and the TOF were enhanced when the atomic ratio of Pt to Pd was changed from 1:0 to about 2:1.

### 3.4. Interfacial Structure Control

**Figure 27:**
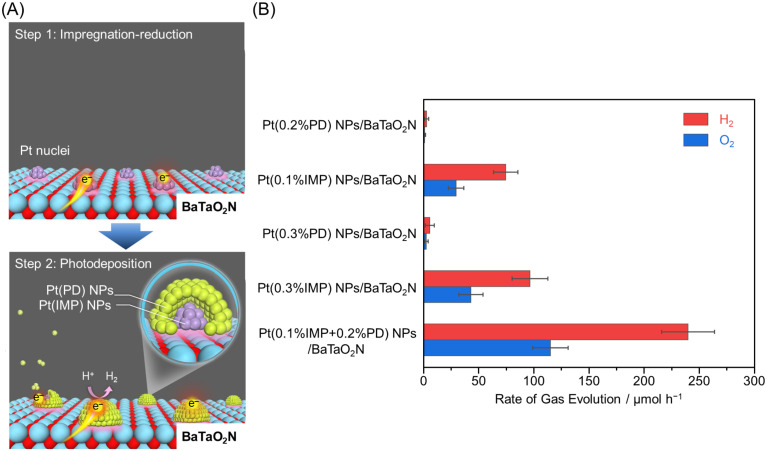
(**A**) Interaction between Pt NPs and BaTaO_2_N photocatalyst. Schematic of sequential Pt NPs cocatalyst deposition on BaTaO_2_N. (**B**) H_2_ and O_2_ evolution rates during Z-scheme water-splitting reaction using Pt NPs/BaTaO_2_N as the HER photocatalyst. For example, Pt (0.2% PD) NPs and Pt (0.1% IMP) NPs represent Pt NPs loaded on BaTaO_2_N by photodeposition and impregnation methods, respectively. Error bars indicate standard deviation of three measurements. Conditions: Pt NP/BaTaO_2_N photocatalyst, 0.1 g; surface-treated WO_3_, 0.15 g; 1 mM of aqueous NaI solution, 150 mL; light source, 300 W Xe lamp (λ ≥ 420 nm) or solar simulator (AM1.5G); irradiation area for solar simulator, 7.6 cm^2^; reaction system, Pyrex top-illuminated vessel connected to the closed gas-circulation system with the periodical evacuation of gas products. Reproduced with permission from reference [225]. Copyright 2021 Springer Nature Limited.

Improving bonding between the cocatalyst and photocatalyst surface is also effective in enhancing the photocatalytic activity. In 2021, Domen, Teshima, and co-workers established a method for loading highly dispersed and uniformly sized HER cocatalysts. In this method, Pt NPs were first loaded on BaTaO_2_N (Figure 7 and Table 3) by impregnation, followed by additional loading of Pt on Pt NPs by photodeposition (Figure 27A) [225]. This sequential loading strongly immobilized Pt NPs on BaTaO_2_N, which facilitated the transfer of photoexcited electrons from the semiconductor to the cocatalyst, and the resulting photocatalyst showed high HER activity (AQY = 6.8 ± 0.5% at 420 nm; Figure 27B and Table 3). The Z-scheme water-splitting reaction using Pt NPs/BaTaO_2_N as the HER photocatalyst and tungsten oxide (WO_3_) as the OER photocatalyst (Figure 4B) showed an AQY = 4.0% (at 420 nm) and STH = 0.24%.

### 3.5. Surface Structure Control

**Figure 28:**
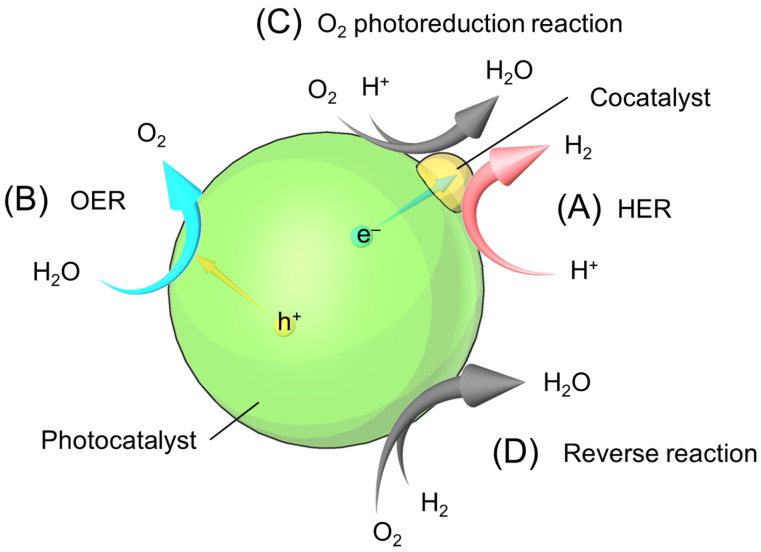
Possible reactions that occur over a water-splitting photocatalyst during the photocatalytic reaction: (A) HER, (B) OER, (C) O_2_ photoreduction reaction, and (D) reverse reaction [79].

The HER proceeds on the noble metal (such as Pt and Rh) NPs cocatalyst. However, when O_2_ is present in the system, the reverse reaction also proceeds in parallel (O_2_ photoreduction and reverse reaction; see Figure 28) on the noble metal NPs cocatalyst. Therefore, to efficiently produce H_2_, the reverse reaction on the noble metal NPs cocatalyst must be suppressed. For this purpose, effective methods are as follows: (i) formation of a reverse-reaction-suppressing layer on the cocatalyst, (ii) formation of a reverse-reaction-suppressing layer on the entire photocatalyst surface, and (iii) giving the cocatalyst itself a reverse-reaction-suppressing function. Such methods are described in the following section.

#### 3.5.1. Formation of Cr_2_O_3_ Shell on Cocatalysts

**Figure 29:**
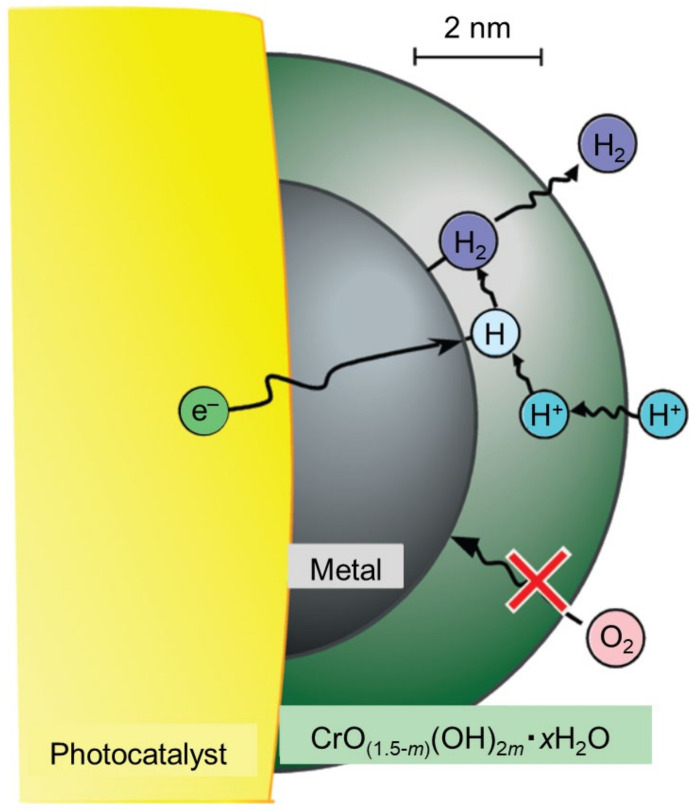
Schematic model of HER on core–shell Cr_2_O_3_/noble-metal NPs system as a cocatalyst for photocatalytic overall water splitting. Reproduced with permission from reference [232]. Copyright 2009 American Chemical Society.

In 2006, Maeda, Domen, and co-workers found that formation of a Cr_2_O_3_ layer on the Rh NPs’ surface (Figure 29) suppressed one of the reverse reactions, namely, the O_2_ photoreduction reaction (Figure 28C) [226,227,228,229,230]. Various experiments on the reaction mechanism showed that H_2_O and H^+^ ions, which are polar molecules, penetrated the Cr_2_O_3_ layer because this layer was hydrated in water, resulting in H_2_ being generated on the Rh surface. On the other hand, O_2_, which is a nonpolar molecule, cannot penetrate the Cr_2_O_3_ layer from the outside, meaning that the formation of this layer prevented the reverse reaction. This inhibition of the reverse reaction has also been observed when other noble NPs, such as Cu, Pd, Pt, and Au, were used as cocatalysts [75,232,233].

#### 3.5.2. Formation of Other Amorphous Metal (Oxy) Hydroxide Layers on the Photocatalyst Surface

The reverse reactions can also be suppressed by loading amorphous oxyhydroxides, such as TiO_2_, Nb_2_O_5_, and Ta_2_O_5_, on the entire surface of the photocatalyst particles [231]. These layers were created by loading peroxide complexes onto the photocatalytic surface by photodeposition. This reaction is a downhill reaction that proceeds more easily than the formation of Cr_2_O_3_ layers described above using an uphill reaction (in this case, photodeposition using CrO_4_^2−^). Therefore, this method can be applied to water-splitting photocatalysts that have weak reducing and oxidizing power owing to their narrow BG.

**Figure 30:**
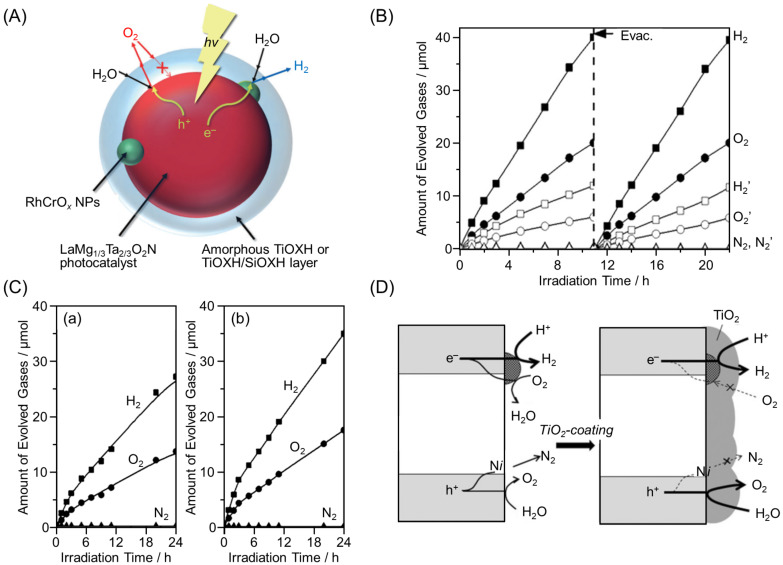
(**A**) Reaction mechanism for water splitting on a surface-coated photocatalyst. (**B**) Gas evolution during water splitting on TiOXH/SiOXH/RhCrO*_x_* NPs/LaMg_1/3_Ta_2/3_O_2_N under UV/Vis-light irradiation (λ > 300 nm; filled symbols) and Vis-light irradiation alone (λ > 420 nm; open symbols). Reaction conditions: Catalyst (0.2 g), reaction solvent (pure water, 250 mL), Xe lamp (300 W); a side-irradiation-type Pyrex reaction vessel was used. (**C**) Time courses of gas evolution on RhCrO*_x_* NPs/LaMg_1/3_Ta_2/3_O_2_N modified by different TiO_2_ coating methods: (**a**) TiO_2_/H_2_O_2_ and (**b**) TTIP/H_2_O_2_. Reaction conditions are the same as (**A**). (**D**) Schematic of the functions of the cocatalyst (RhCrO*_x_*) and the coating layer (TiO_2_). N, unstable surface nitrogen; N*_i_*, nitrogen at the interface. Reproduced with permission from references [234,235]. Copyright 2015 Wiley-VCH and 2016 Wiley-VCH.

For example, in 2015, Takata, Domen, and co-workers mixed TiO_2_ and RhCrO*_x_* NPs/LaMg_1/3_Ta_2/3_O_2_N (Mg = magnesium) in hydrogen peroxide (H_2_O_2_) solution and irradiated them with light to form a core–shell structure in which TiOXH (OXH = oxyhydroxide) covered the entire surface of the semiconductor photocatalyst (LaMg_1/3_Ta_2/3_O_2_N) and the cocatalyst particles (RhCrO*_x_* NPs) (Figure 30A). The authors found that this suppressed the O_2_ reduction reaction in the as-obtained TiOXH/RhCrO*_x_* NPs/LaMg_1/3_Ta_2/3_O_2_N [234]. The authors also succeeded in forming a double-coating layer consisting of SiOXH and TiOXH on the surface of LaMg_1/3_Ta_2/3_O_2_N using a similar preparation method (Figure 30A). In this case, the amount of H_2_ and O_2_ evolution increased linearly with irradiation time (Figure 30B). Double coating resulted in the formation of a more uniform layer. Additionally, when SiOXH was mixed into the layer, the hydrophilicity of the layer increased, further inhibiting the permeation of O_2_ molecules. These factors resulted in more effective suppression of the reverse reaction.

In 2016, these authors also reported the effect of the type of precursor on the function of the photocatalyst. Changing the precursor from TiO_2_ to titanium tetraisopropoxide (TTIP) was found to enhance the water-splitting activity of the resulting photocatalyst 1.4 times (Figure 30C) [235]. This paper also reported that the formation of TiOXH layers enhanced the stability of LaMg_1/3_Ta_2/3_O_2_N during photocatalytic reactions (Figure 30D).

#### 3.5.3. Formation of Solid Solution (Rh–Cr, Rh–Zr) Oxide Cocatalysts

In 2006, Maeda and Domen showed that suppression of the reverse reaction due to Cr_2_O_3_ also occurred when the solid-solution structure was formed instead of the core–shell structure [236,237,238,239,240]. Such a suppression effect of Rh_2−*x*_Cr*_x_*O_3_ NPs on the reverse reaction was also observed in our study on UV-light-driven photocatalysis (Figure 19) [77].

Furthermore, long-term light irradiation of the obtained photocatalyst led to the dissolution of Cr^6+^ ions [284]. This results in decreased catalytic activity and is considered to have an impact on environmental destruction and health hazards. Therefore, Saruyama, Teranishi, and co-workers worked to create cocatalysts with elements other than Cr that have similar effects. As a result, in 2020, these authors found that the RhZrO*_x_* solid-solution NPs cocatalyst, which contained Zr and Rh, also had the ability to suppress the reverse reaction [241]. Although UV-light-driven aluminum (Al)-doped SrTiO_3_ (SrTiO_3_:Al) [285] was used as a photocatalyst in this study, future studies are expected to be conducted on Vis-light-driven water-splitting photocatalysts using the same NPs as a cocatalyst.

### 3.6. Charge-State Control

**Figure 31:**
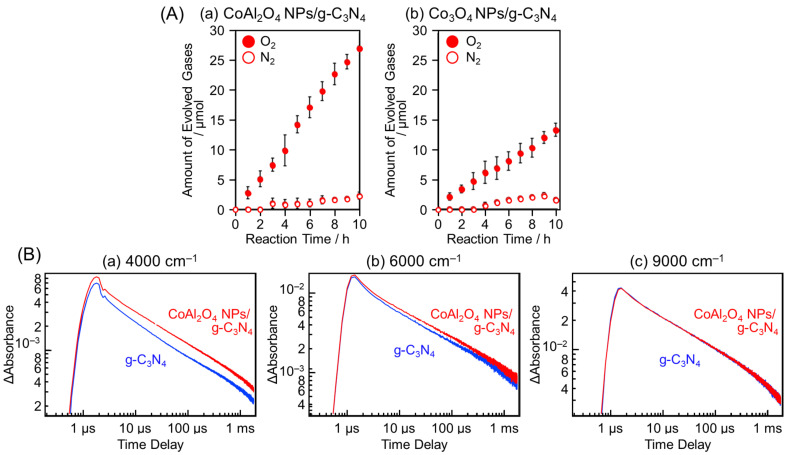
(**A**) Time course of photocatalytic O_2_ evolution over g-C_3_N_4_ loaded with (**a**) CoAl_2_O_4_ (3.0 wt%) NPs and (**b**) Co_3_O_4_ (1.5 wt%) NPs cocatalysts. Reaction conditions: catalyst, 50 mg; La_2_O_3_, 200 mg; reactant solution, aqueous AgNO_3_ (10 mM, 140 mL); light source, 300 W Xe lamp with cut-off filter (L42). (**B**) Decay curves for transient absorption intensity at different wavenumbers for g-C_3_N_4_ and CoAl_2_O_4_ (3.0 wt%) NPs/g-C_3_N_4_. The decay kinetics of the more reactive electrons recorded at 4000 cm^−1^ (**a**) became slower after modification of CoAl_2_O_4_ NPs on the surface of g-C_3_N_4_, indicating that the population of surviving electrons was increased by loading with CoAl_2_O_4_ NPs. However, such a slow decay was not clear at 6000 cm^−1^ (**b**), and not observed at 9000 cm^−1^ (**c**). These results strongly suggest that photogenerated holes in g-C_3_N_4_ could move to the loaded CoAl_2_O_4_ NPs, thereby increasing the electron population. Reproduced with permission from reference [242]. Copyright 2020 American Chemical Society.

The function of the cocatalyst also depends on the charge state of the metal contained in the cocatalyst. Cobalt oxide (CoO*_x_*) is often used as an OER cocatalyst owing to its high efficiency, low cost, and earth abundance. Yamakata, Maeda, and co-workers reported in 2020 that g-C_3_N_4_ loaded with Co^2+^-based spinel-type CoAl_2_O_4_ NPs cocatalysts (5–20 nm) showed higher OER activity compared with g-C_3_N_4_ loaded with spinel-type Co_3_O_4_ NPs cocatalysts (5–20 nm) consisting of a mixture of Co^2+^ and Co^3+^ (Figure 31A) [242]. The results of transient absorption spectroscopy showed that the stronger hole-trapping effect in CoAl_2_O_4_ NPs compared with Co_3_O_4_ cocatalysts caused this phenomenon (Figure 31B).

**Figure 32:**
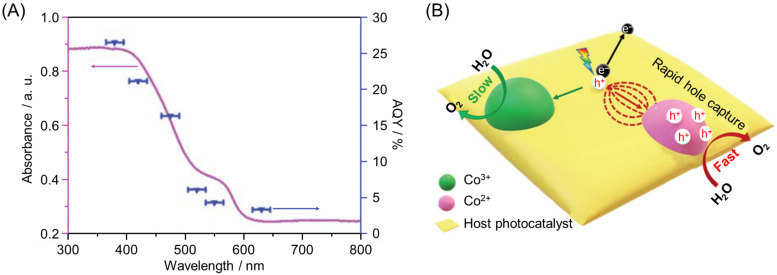
(**A**) Absorption spectrum and wavelength-dependent AQY of photocatalytic O_2_ evolution for CoO*_x_* (Co^2+^) NPs/TaON. (**B**) Proposed mechanism of the photocatalytic water-oxidation process of CoO*_x_* NPs/TaON, indicating accelerated hole transportation and reaction on the Co^2+^ species. Reproduced with permission from reference [243]. Copyright 2021 The Royal Society of Chemistry.

Similar results have been reported by Liu, Yang, and co-workers. In 2021, these authors successfully loaded Co^2+^-based CoO*_x_* cocatalysts (~2.5 nm) or Co^3+^-based CoO*_x_* cocatalysts (~2.6 nm) on TaON photocatalysts using a photochemical metal–organic deposition (PMOD) method [243]. Photocatalytic studies showed that Co^2+^-based CoO*_x_* cocatalysts were 1.6 times more effective for OER than Co^3+^-based CoO*_x_* cocatalysts and that TaON loaded with Co^2+^-based CoO*_x_* cocatalysts had an AQY of 21.2% (at 420 ± 15 nm; Figure 32A and Table 3). Photoelectrochemical reactions and photoluminescence (PL) measurements indicated that Co^2+^ species played an important role in accelerating charge separation and transport (Figure 32B).

## 4. Separation of Each Reaction Site

**Figure 33:**
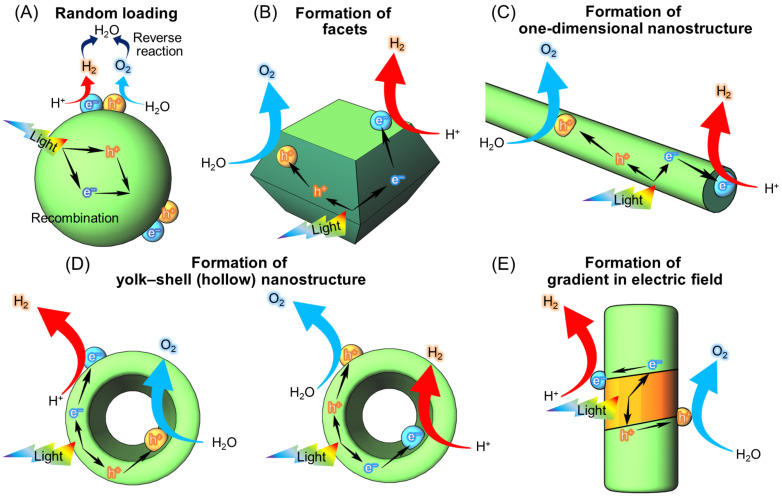
Schematic illustrations of the functionalization of photocatalysts by separating each reaction site. (**A**) Random loading of cocatalysts, (**B**) formation of facets that both excited electrons and holes can easily reach (Section 4.1), (**C**) formation of one-dimensional nanostructure (Section 4.2), (**D**) formation of yolk−shell (hollow) nanostructure (Section 4.3), and (**E**) formation of gradient in electric field (Section 4.4).

To create highly active water-splitting photocatalysts, exciton recombination must be suppressed [286]. Therefore, in recent years, the development of water-splitting photocatalysts in which the excited electrons and holes are spatially separated has been promoted, such that the reduction and oxidation reactions can proceed on different surfaces. One approach is to load both the HER and OER cocatalysts on the photocatalyst (dual-cocatalyst loading). With such a dual-cocatalyst loading, the excited electrons and holes transfer toward the respective cocatalysts. As a result, they can be spatially separated, and the water-oxidation and water-reduction reactions can proceed on different surfaces of the photocatalyst. However, when loading of the dual-cocatalyst is conducted randomly, the two types of cocatalysts cannot be spatially separated, thereby causing the recombination of excitons and reverse reactions to proceed, which might decrease the photocatalytic activity (Figure 33A). Therefore, loading cocatalysts onto suitable sites for each reaction is essential. The following methods can somewhat avoid the exciton recombination and reverse reactions proceeding: (i) forming facets that both excited electrons and holes can easily reach (Section 4.1; Figure 33B) [287,288]; (ii) forming one-dimensional structures (Section 4.2; Figure 33C) [289,290]; (iii) forming a yolk–shell structure (Section 4.3; Figure 33D) [291,292,293]; and (iv) forming a built-in electric field in the band structure (Section 4.4; Figure 33E) [141,155]. For some of the semiconductor photocatalysts described in this section, the appropriate cocatalysts, available reactions (OWSR, HER, or OER; Figure 4), activities, and references are summarized in Table 4 [287,288,289,290,291,292,293].

### 4.1. Forming Facets That Both Excited Electrons and Holes Can Easily Reach

**Figure 34:**
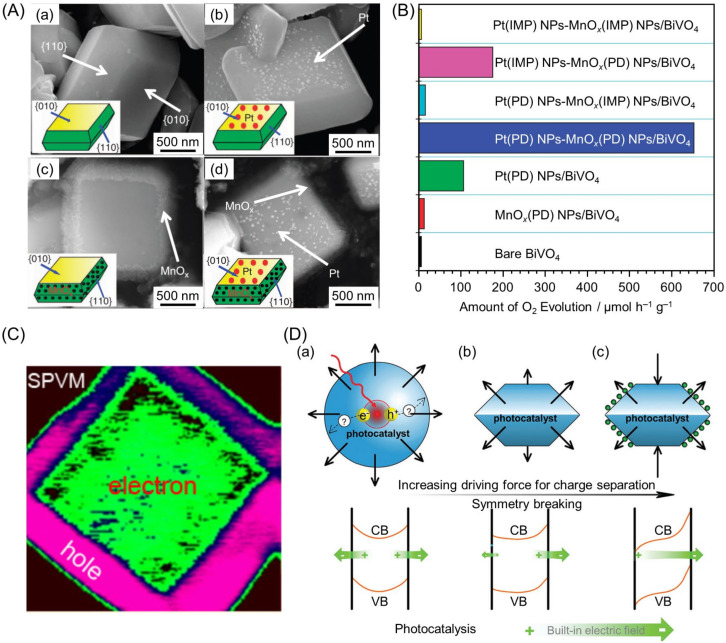
(**A**) SEM images of BiVO_4_ with and without metal/metal oxide NPs. (**a**) Bare BiVO_4_, (**b**) Pt(PD) NPs/BiVO_4_, (**c**) MnO*_x_*(PD) NPs/BiVO_4_, and (**d**) Pt(PD) NPs-MnO*_x_*(PD) NPs/BiVO_4_. Contents of the deposited metals/metal oxides are all 5 wt%. (**B**) Amount of O_2_ evolved over each photocatalyst. Reaction conditions: NaIO_3_ aqueous solution (150 mL, 0.02 M), Xe lamp (300 W, λ ≥ 420 nm), top irradiation, reaction time of 1 h. (IMP, impregnation method; PD, photodeposition method; contents of the deposited cocatalysts are all 0.1 wt%). (**C**) Spatial distribution of the surface photovoltage signals. Pink and green colors correspond to holes and electrons separated toward the external surface, respectively. (**D**) Schematic illustrations showing the driving force for charge separation in photocatalyst particles with (**a**) symmetric built-in electric fields, (**b**) anisotropic built-in electric fields on different facets, and (**c**) an asymmetric cocatalyst assembly. Reproduced with permission from references [287,295,296]. Copyright 2013 Springer Nature Limited, 2017 American Chemical Society, and 2018 The Royal Society of Chemistry.

Some crystallized semiconductor photocatalysts might have facets that are more suitable for reduction or oxidation reactions [294]. For example, *m*-BiVO_4_ has a decahedral structure consisting of (010) and (110) facets, where reduction reactions selectively occur on the (010) facets and oxidation reactions on the (110) facets. In 2013, Li, Zhang, and co-workers succeeded in selectively loading Pt NPs as an HER cocatalyst on the (010) facet and MnO*_x_*(PD) NPs as an OER cocatalyst on the (110) facet using the photodeposition method (Figure 34A) [287]. As-obtained Pt(PD) NPs-MnO*_x_*(PD) NPs/BiVO_4_ showed a much higher OER rate compared with the photocatalyst with cocatalysts randomly loaded using the impregnation method (Figure 34B). The high OER rate of Pt(PD) NPs-MnO*_x_*(PD) NPs/BiVO_4_ obtained using the photodeposition method was attributed to the excited electrons and holes being transferred to different crystal facets and separated efficiently (Figure 34C) [295]. In 2017, Fan, Dittrich, Li, and co-workers also showed that the migration direction of electrons and holes strongly depended on the built-in electric field present in the space charge region of each facet; therefore, controlling the shape of photocatalytic particles and increasing the difference in the internal electric field between each facet can enhance the separation of electrons and holes (Figure 34D) [296]. These authors also found that an asymmetrical array of cocatalysts further enhances the difference in the built-in electric field at the surface (Figure 34D).

**Figure 35:**
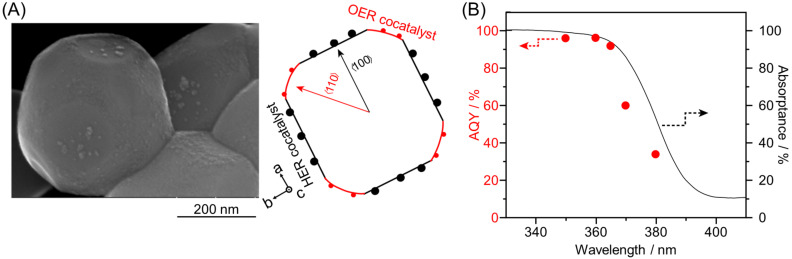
(**A**) SEM and schematic images of SrTiO_3_:Al loaded with various cocatalysts. (**B**) UV–Vis diffuse reflectance spectrum of bare SrTiO_3_:Al (black solid line) and wavelength dependence of AQY during water splitting on Cr_2_O_3_ (0.05 wt%)/Rh (0.1 wt%) NPs-CoOOH (0.05 wt%) NPs/SrTiO_3_:Al (red symbols). Reproduced with permission from reference [288]. Copyright 2020 Springer Nature Limited.

The flux method is extremely effective for the synthesis of photocatalysts with such specific facets. In the flux method, raw powder is heated and dissolved in the flux, and then crystals are precipitated by the increase in supersaturation caused by cooling and evaporation of the flux. This method has been used in the synthesis of metal oxides such as K_4_Nb_6_O_17_ (K = potassium) [297,298,299,300], KNb_3_O_8_ [301,302], Na_2_Ti_6_O_13_ [303,304,305], and SnNb_2_O_6_ [306,307]. In 2020, Takata, Domen, and co-workers controlled the particle shape of SrTiO_3_:Al using a flux method and subsequently successfully loaded HER and OER cocatalysts selectively onto specific crystal surfaces of SrTiO_3_:Al particles (Figure 35A) [288]. In the structure, the photoexcited electrons and holes were selectively transferred to the HER and OER cocatalysts, respectively, and the recombination of electrons and holes, which caused the QY to decrease in conventional photocatalysts, was almost completely suppressed. Therefore, this photocatalyst showed an AQY of 96% (at 350–360 nm) (Figure 35B). In this study, the UV-light-driven semiconductor SrTiO_3_:Al was used as a water-splitting photocatalyst. In future studies, such a method is expected to be applied to create highly functional Vis-light-driven water-splitting photocatalysts.

### 4.2. Formation of One-Dimensional (1D) Nanostructures

**Figure 36:**
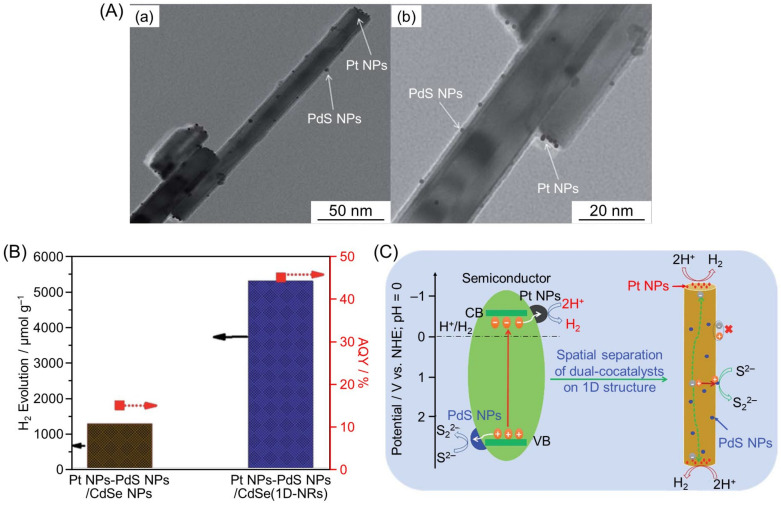
(**A**) TEM images of Pt NPs-PdS NPs/CdSe 1D nanorods (CdSe(1D-NRs)). Images (**a**) and (**b**) are at different magnifications. (**B**) Comparative H_2_ evolution of Pt NPs-PdS NPs/CdSe(1D-NRs) and Pt NPs-PdS NPs/CdSe NPs. Conditions: catalyst (100 mg), Na_2_S–Na_2_SO_3_ aqueous solution (100 mL, 0.1 M), and Xe lamp light source (300 W) with an optical filter (λ > 420 nm). (**C**) Scheme of the proposed mechanism for photocatalytic H_2_ evolution on CdSe(1D-NRs) with spatial separation of dual cocatalysts. Reproduced with permission from reference [289]. Copyright 2019 The Royal Society of Chemistry.

In some cases, the formation of 1D nanostructures can efficiently separate electrons and holes spatially. In 2019, Li and co-workers successfully loaded Pt NPs (HER cocatalyst) on the tip and PdS NPs (OER cocatalyst) on the side of CdSe 1D nanorods (CdSe(1D-NRs)) by photodeposition (Pt NPs-PdS NPs/CdSe(1D-NRs); Figure 36A) [289]. The as-obtained Pt NPs-PdS NPs/CdSe(1D-NRs) photocatalyst showed a HER activity more than 20 times higher than that of the conventional Pt NPs-PdS NPs/CdSe NPs photocatalyst (AQY = ~45% (at 420 nm); Figure 36B). PL measurements showed that these excellent photocatalytic performances were attributed to the decrease in exciton recombination in the CdSe(1D-NRs) caused by the spatial separation of the cocatalysts (Figure 36C). In this photocatalyst, the excited electrons migrate along the long axis to the tip, while the holes migrate to the sides, causing the effective separation of electrons and holes.

**Figure 37:**
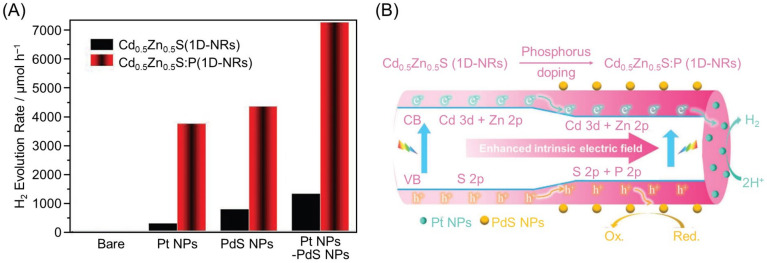
(**A**) Photocatalytic H_2_ evolution performance of different samples. Conditions: catalyst, 0.1 g; 0.1 M of Na_2_S–Na_2_SO_3_ aqueous solution, 100 mL; Xe lamp light source (300 W) with an optical filter (λ > 420 nm). (**B**) Proposed photogenerated electron–hole transfer mechanism for P-doped CZS NRs to induce a localized intrinsic electric field for spatial separation of redox cocatalysts. Reproduced with permission from reference [290]. Copyright 2020 Wiley-VCH.

Furthermore, in 2020, Li and co-workers reported that P-doping the surface of the Cd_0.5_Zn_0.5_S(1D-NRs) photocatalyst (Cd_0.5_Zn_0.5_S(1D-NRs):P) enhances electron and hole transfer, producing a Vis-light-driven water-splitting photocatalyst with a high QY [290]. These authors successfully improved the HER activity of the Cd_0.5_Zn_0.5_S(1D-NRs) photocatalyst under Vis-light irradiation by more than two orders of magnitude. They also succeeded in improving the AQY to 89% (at 420 nm) by loading Pt NPs and PdS NPs cocatalysts on the tips and sides of the obtained Cd_0.5_Zn_0.5_S:P(1D-NRs), respectively (Figure 37).

### 4.3. Formation of Yolk–Shell (Hollow) Nanostructure

**Figure 38:**
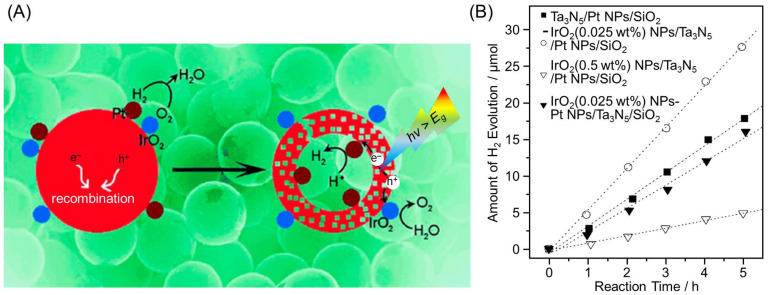
(**A**) Design principle for a yolk–shell Ta_3_N_5_ photocatalyst loaded with two separate cocatalysts as effective charge collectors for water splitting. (**B**) H_2_-evolution activities on yolk–shell Ta_3_N_5_ photocatalysts with separated and mixed Pt NPs and IrO_2_ NPs cocatalysts. Reaction conditions: Ta_3_N_5_ catalyst, 0.032 g; methanol, 20 mL; H_2_O, 80 mL; 300 W Xe lamp with an L42 cutoff filter; top irradiation. Reproduced with permission from reference [291]. Copyright 2013 Wiley-VCH.

**Figure 39:**
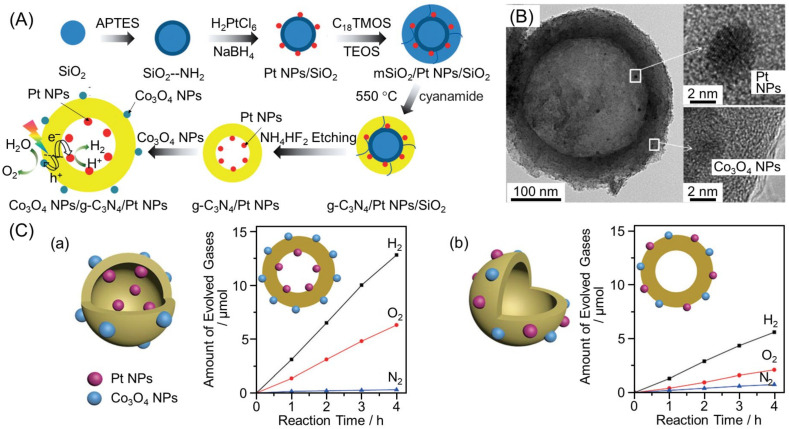
(**A**) Illustration of the preparation of Co_3_O_4_ NPs/g-C_3_N_4_/Pt NPs composites. APTES, C18-TMOS, and TEOS represent 3-aminopropyl triethoxysilane, *n*-octadecyltrimethoxysilane, and tetraethoxysilane, respectively. (**B**) TEM and HR-TEM images of Co_3_O_4_ NPs/g-C_3_N_4_/Pt NPs samples. (**C**) Time courses of photocatalytic evolution of H_2_ and O_2_ over (**a**) Co_3_O_4_ NPs/g-C_3_N_4_/Pt NPs and (**b**) Co_3_O_4_ NPs-Pt NPs/g-C_3_N_4_ under UV-light irradiation (λ > 300 nm). Reproduced with permission from reference [292]. Copyright 2016 Wiley-VCH.

**Figure 40:**
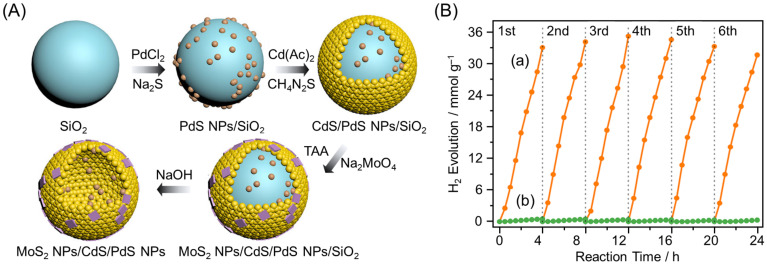
(**A**) Schematic illustration of the synthetic process for MoS_2_ NPs/CdS/PdS NPs heterostructures. (**B**) Cycle stability of H_2_ evolution from 0.1 M of Na_2_S–Na_2_SO_3_ aqueous solution under Vis-light irradiation (420–780 nm) using (a) MoS_2_(7.5 wt%) NPs/CdS/PdS(1.5 wt%) NPs hollow spheres and (b) CdS hollow spheres (each cycle is 4 h). Reproduced with permission from reference [293]. Copyright 2021 Wiley-VCH.

Photocatalysts with suppressed recombination and reverse reactions can be developed by loading HER and OER cocatalysts on the inside and outside of the yolk–shell nanostructure, respectively (Figure 33D). Indeed, in 2013, Domen and co-workers succeeded in significantly enhancing the HER activity by forming Ta_3_N_5_ yolk–shell nanostructures using SiO_2_ as a template and selectively loading Pt NPs (HER cocatalyst) and IrO_2_ NPs (OER cocatalyst) onto the inside and outside surfaces, respectively (Figure 38) [291]. In 2016, Wang and co-workers also successfully formed g-C_3_N_4_ hollow spheres using aminated SiO_2_ as a template and loaded HER cocatalysts (Pt NPs) and OER cocatalysts (Co_3_O_4_ NPs) onto the inside and outside surfaces, respectively (Figure 39A,B). The obtained photocatalyst showed overall water splitting with the molar ratio of H_2_ to O_2_ of 2:1 under UV-light irradiation (Figure 39C(a)) [292]. When both Pt NPs and Co_3_O_4_ NPs were loaded onto the outside surface of g-C_3_N_4_, a decrease in water-splitting activity due to decreased O_2_ evolution was observed (Figure 39C(b)). This indicated that separately loading each cocatalyst onto the inside and outside of the yolk–shell nanostructure is extremely important to obtain high activity. In 2021, Wang and co-workers also succeeded in creating photocatalysts with HER cocatalysts (MoS_2_ NPs) and OER cocatalysts (PdS NPs) loaded onto the outside and inside of CdS hollow spheres, respectively (Figure 40A) [293]. As exciton recombination was greatly suppressed, the obtained photocatalyst showed 115 times higher HER activity compared with CdS NPs (Figure 40B).

### 4.4. Formation of Built-in Electric Field in Band Structure

**Figure 41:**
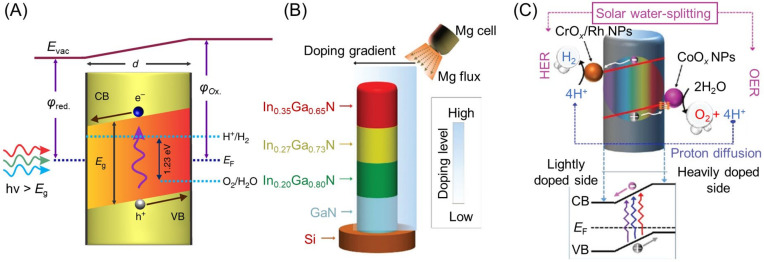
(**A**) Structural and optical properties of InGaN photochemical diode. Energy-band representation of the proposed photochemical diode (PCD) with radial thickness “*d*” showing the built-in electric field (band-bending) that separates the excitons (electron and hole) and drives them towards the opposite cathode and anode surfaces. In contrast to a conventional p–n PCD, only a single photon absorption is required to generate one active electron–hole pair to participate in the redox reaction (such as a Schottky-type photochemical diode). (**B**) Schematic of the quadruple-band InGaN nanowire. The p-type dopant originating from the tilted Mg effusion cell (relative to the nanowire orientation) leads to the Mg-doping gradient profile in the lateral direction of the nanowire. (**C**) The InGaN nanowire can introduce a built-in electric field for efficient charge carrier separation and extraction for water redox reactions. Reproduced with permission from references [141,155]. Copyright 2018 Springer Nature Limited and 2019 The Royal Society of Chemistry.

In 2018, Mi and co-workers proposed a photochemical diode structure in which the excitons generated by photoexcitation were directed to different active sites (Figure 41A) [155]. The proposed structure consisted of vertically aligned InGaN nanowires, with the active sites of HER and OER clearly defined. Photogenerated electrons and holes were instantly separated by the electric field formed perpendicularly to the nanosheet (Figure 41A), which suppressed recombination and reverse reactions at the surface and in the bulk. Specifically, the authors introduced an in-built electric field along the lateral direction of the nanowires by varying the amount of Mg doping (Figure 41B). Photoexcited electrons transferred to the less Mg-doped side and proceeded with HER, while holes transferred to the more Mg-doped side and proceeded with OER (Figure 41C). When the HER cocatalyst (Cr_2_O_3_/Rh NPs) and OER cocatalyst (CoO*_x_* NPs) were loaded on this photocatalyst, the STH reached 5.2% (Table 2) [141].

## 5. Summary

This review summarized representative studies of Vis-light-driven water-splitting photocatalysts. This summary clarified the following points regarding the fabrication and functionalization of Vis-light-driven water-splitting photocatalysts.

(1) To develop Vis-light-driven water-splitting photocatalysts, modifying the band structure of stable metal oxide photocatalysts is effective. The main methods are (i) shifting the energy position of the VBM to the negative-potential side by anion or metal cation substitution, (ii) forming impurity levels in the BG by doping, (iii) narrowing the BG by solid solution, and (iv) shifting the entire band structure to the negative-potential side by a reduction in the size of semiconductor particles.
(2) Vis-light-driven water-splitting photocatalysts can also be created by (i) forming multiband-InGaN nanowires, (ii) utilizing g-C_3_N_4_, and (iii) forming MOF/COF connections.
(3) Controlling the particle size, chemical composition, morphology, interfacial structure, surface structure, and charge state of the cocatalyst is extremely effective at enhancing the functionality of the photocatalyst.
(4) Forming crystal facets on the photocatalyst surface that excited electrons and holes can easily reach, one-dimensional NR structures, yolk–shell structures, and a built-in electric field in the band structure effectively promote charge separation and suppress recombination, resulting in a high QY.

Sharing these findings with readers is expected to further accelerate the development and practical application of Vis-light-driven water-splitting photocatalysts.

## 6. Outlook

For the practical application of Vis-light-driven water-splitting photocatalysts, much effort is expected to be devoted to the following research areas:

(1) **Identifying a simple synthesis method for Vis-light-driven water-splitting photocatalysts.** Although several materials have been developed for Vis-light-driven water-splitting photocatalysts, metal (oxy)nitrides and metal (oxy)sulfides are the most attractive materials for OWSR in terms of QY. However, synthesis methods for these photocatalysts have been established only under specific atmospheres. Therefore, only a few research groups with synthesis experience have been able to study these photocatalysts. In the future, simpler methods for synthesizing metal(oxy)nitrides and metal(oxy)sulfide water-splitting photocatalysts are expected to be developed. If realized, more research groups will be able to participate in research on the functionalization of these materials, which is expected to bring their practical application closer to fruition.
(2) **Enhancement of exciton separation efficiency.** To obtain highly active water-splitting photocatalysts, the exciton separation efficiency must be enhanced [288]. In future, fluorescence lifetime and transient absorption spectroscopic measurements [308,309,310,311,312,313,314] are expected to be conducted for a number of photocatalysts to gain a deeper understanding of the influence of the photocatalyst substrate, cocatalyst, and interfacial structure between them on the charge separation efficiency.
(3) **Structural analysis of loaded cocatalysts.** In order to understand structure–property relationships, it is essential to gain a deeper understanding of the geometric structure of the loaded cocatalysts, especially during the reaction. Therefore, in the future, the geometric structure of loaded cocatalysts is expected to be directly observed using aberration-corrected TEM [315] or scanning TEM. Furthermore, the geometric structure observed under electron irradiation in a vacuum is not necessarily the same as the geometric structure during the water-splitting reaction. Therefore, operando measurements using X-ray absorption fine-structure analysis and other techniques [316] are expected to be applied in photocatalyst studies, which will provide a deeper understanding of the geometric structure during the water-splitting reaction than available at present.
(4) **Theoretical calculation for real system.** Theoretical calculations are also useful for developing highly functional water-splitting photocatalysts. Indeed, previous theoretical calculations have clarified the adsorption state of water molecules [317,318,319], the rate-limiting step of the reaction [320,321], and the exciton transfer process at the interface between water molecules and photocatalysts [322,323]. However, in most of these studies, theoretical calculations have been performed on simplified models of real systems, and simulations have been performed on a timescale (<1 μs) shorter than the actual reaction time (10–900 μs). In future, calculations are expected to be performed on longer timescales for real systems, which will provide a deeper understanding of the photocatalytic reaction process.
(5) **Construction of practical application system.** Photocatalysts with an STH exceeding 10% need to be developed for practical use in water splitting. Furthermore, a system for the social implementation of water-splitting photocatalysts must be constructed simultaneously. Accordingly, Domen and co-workers have recently succeeded in constructing a H_2_-production system using photocatalytic panels. However, at present, remaining challenges include the fabrication cost of photocatalytic panels and the performance of the module that separates the evolved gases (H_2_ and O_2_) [324,325]. In future, industry–academia collaborative research is anticipated to be conducted more extensively, which will lead to these challenges being overcome.

## Figures and Tables

**Table 1:** BG, Effective cocatalysts, possible reaction, and efficiency of Vis-light-driven water-splitting photocatalysts introduced in Section 2.1.

Photocatalyst	BG /eV	Cocatalyst (/wt%)	Possible Reaction *^a^*	Efficiency *^b^*	Ref.
Ag(I)-Ga_2_In_3_S_8_	2.3	Rh (0.5)	HER	AQY = 15% (at 460 nm)	[116]
Ag(I)-K_2_SrTa_2_O_7_	2.8	Bare	OER	ER = 3.0 µmol h^−1^ (>420 nm)	[117]
Ag(I)-Li_2_SrTa_2_O_7_	2.8	Bare	OER	ER = 4.0 µmol h^−1^ (>420 nm)	[117]
Bi_4_NbO_8_Cl	2.4	Pt (0.5)	OER (pH = 2)	AQY = 0.4% (at 420 nm)	[110]
Bi_4_TaO_8_Br	2.5	RuO_2_ (0.7)	OER	AQY > 20% (at 420 nm)	[111]
Bi_4_NbO_8_Br	2.5	N/A	N/A	N/A	[109]
BiOCl	3.4	N/A	N/A	N/A	[109]
BiOBr	2.8	N/A	N/A	N/A	[109]
BiOI	1.8	N/A	N/A	N/A	[109]
BiVO_4_	2.4	Bare	OER	AQY = 9.0% (at 450 nm)	[112]
*m*–BiVO_4_:In,Mo	2.5	RuO_2_ (3.0)	OWSR (pH = 7)	AQY = 3.2% (at 420–800 nm)	[128]
CdS-P	1.8	CoP (5.0)	HER	N/A	[107]
CoO NPs	2.6	Bare	OWSR	STH = ~5.0% (AM1.5G)	[134]
Cu(I)-Ga_2_In_3_S_8_	1.9	Rh (1.5)	HER	AQY = 15% (at 560 nm)	[116]
Cu(I)-K_2_La_2_Ta_3_O_10_	2.0	Ru (0.3)	HER	ER = 45 µmol h^−1^ (>420 nm)	[117]
Cu(I)-K_2_SrTa_2_O_7_	2.1	Ru (0.3)	HER	ER = 66 µmol h^−1^ (>420 nm)	[117]
GaN:ZnO	2.6	RuO_2_ (5.0)	OWSR (pH = 3)	AQY = 0.14% (at 300–480 nm)	[129]
		Rh_2–*x*_Cr*_x_*O_3_ (Rh: 1.0, Cr: 1.5)	OWSR (pH = 4.5)	AQY = 5.9% (at 420–440 nm)	[130]
La_5_Ti_2_Cu_0.9_Ag_0.1_S_5_O_7_ :Ga	1.8	Cr_2_O_3_/Rh	OWSR (pH = 10) (-Au-BiVO_4_)	AQY = 4.9% (>420 nm) STH = 0.11%	[131]
La_5_Ti_2_CuS_5_O_7_	1.9	Pt (0.3) -NiS (1.0)	HER (pH = 10)	AQY = 1.8% (at 420 ± 10 nm)	[105,106]
La_5_Ti_2_Cu(S_1__–_*_x_*Se*_x_*)_5_O_7_	1.6–1.9	NiS (0.5 or 1.0)	HER (pH = 12)	N/A	[132]
La_2_Ta_2_ZrS_2_O_8_	2.4	IrO_2_ (0.5)	OER (pH = 13)	N/A	[102]
Cu(I)-NaTaO_3_	2.0	Ru (0.3)	HER	AQY = 0.18% (>420 nm)	[114]
Ag(I)-Na_2_W_4_O_13_	2.9	N/A	OER (pH = 2.4)	ER = 0.9 µmol h^−1^ (>420 nm)	[118]
Sm_2_Ti_2_S_2_O_5_	2.1	Pt (1.0)	HER (pH = 8–9)	ER = 22 µmol h^−1^ (440 nm ≤ λ ≤ 650 nm)	[100,109]
			OER (pH = 10)	ER = 22 µmol h^−1^ (440 nm ≤ λ ≤ 650 nm)	[101]
SrTiO_3_:Rh	1.7	Pt (0.1)	HER	AQY = 5.2% (at 420 nm)	[119]
SrTiO_3_:Sb,Rh	N/A	InO_2_ (3.0)	HER (pH = 3)	AQY = 0.1% (at 420 nm)	[120]
TaON	2.4	Pt (3.0)	HER	AQY = 0.2% (at 420 nm)	[92,94]
		Pt (3.0)	OER (pH = ~8)	AQY = 34% (at 420 nm)	[94]
		Ru (0.05)	HER	ER = 120 µmol h^−1^ (>420 nm)	[103,107]
		Cr_2_O_3_ (2.5) /RuO*_x_*(3.0) -IrO_2_ (4.0)	OWSR	AQY < 0.1% (at 420 nm)	
Ta_3_N_5_	2.1	Pt (3.0)	HER	AQY = 0.1% (at 420–600 nm)	[92,97]
		Cr_2_O_3_ (2.5) /RuO*_x_*(3.0) -IrO_2_ (4.0)	OER (pH = 8.5)	AQY = 10% (at 420–600 nm)	[98]
Y_2_Ti_2_O_5_S_2_	1.9	Cr_2_O_3_ (1.5) /Rh (2.0) -IrO_2_ (0.3)	OWSR (pH = 8.5)	AQY (H_2_) = 5.3 ± 0.3% (at 420–480 nm) AQY (O_2_) = 2.3 ± 0.1% (at 420–480 nm)	[103]

*^a^* OER, HER, and OWSR represent the oxygen evolution reaction, hydrogen evolution reaction, and overall water-splitting reaction, respectively. *^b^* AQY, ER, STH, and AM1.5G represent the apparent quantum yield, evolution rate, solar-to-hydrogen conversion efficiency, and global standard solar spectrum (AM1.5G), respectively.

**Table 2:** BG, Effective cocatalysts, possible reaction, and efficiency of Vis-light-driven water-splitting photocatalysts introduced in Section 2.2.

Photocatalyst	BG /eV	Cocatalyst (/wt%)	Possible Reaction *^a^*	Efficiency *^b^*	Ref.
COF (TpPa-1-COF) /MOF (NH_2_-UiO-66)	COF: 2.0 MOF: 2.9	Pt (3.0)	HER	ER = 23.4 mmol g^−1^ h^−1^ (>420 nm)	[153]
COF (TFPT–DETH) /MOF (NH_2_-UiO-66)	COF: 2.8 MOF: 2.8	Pt (7.5)	HER (pH = 7.4)	AQY = 1.1% (at 420 nm)	[154]
g-C_3_N_4_:O	3.0	Pt (3.0)	HER	ER = ~189.3 µmol h^−1^ (>400 nm)	[142]
g-C_3_N_4_:P	2.5	Pt (1.0)	HER	ER = 2610.8 µmol h^−1^ g^−1^ (≥420 nm)	[143]
g-C_3_N_4_:S	2.9	Pt (6.0)	HER	N/A	[144]
	2.6	Pt (3.0)	HER (pH = 8.5)	AQY = 5.8% (at 440 nm)	[145]
g-C_3_N_4_:B	2.6	Pt (1.0)	HER	ER = 94 µmol h^−1^ (>400 nm)	[146]
g-C_3_N_4_:F	2.6	Pt (3.0)	HER	TOF = 0.125 h^−1^ (>420 nm)	[147]
g-C_3_N_4_:I	2.4	Pt (3.0)	HER (pH = 7)	AQY = 3.0% (at 420 nm)	[148]
g-C_3_N_4_:Cu^2+^	2.3	N/A	N/A	N/A	[150]
g-C_3_N_4_:Fe^3+^	2.5	Bare	HER	ER = 536 µmol h^−1^ (>420 nm)	[149]
g-C_3_N_4_:Zn^2+^	2.7	Pt (0.5)	HER	AQY = 3.2% (at 420 nm)	[151]
g-C_3_N_4_ ultra-thin nanosheet	3.0	Pt (1.4)	OWSR	AQY = 0.23% (at 400 nm)	[152]
GaN	3.4	CrO*_x_*/Rh	OWSR (pH = ~7) (Quadruple-band nanowire)	STH = 5.2% (AM1.5G)	[141]
In_0.35_Ga_0.65_N	2.1				
In_0.27_Ga_0.73_N	2.4				
In_0.20_Ga_0.80_N	2.6				

*^a^* OER, HER, and OWSR represent the oxygen evolution reaction, hydrogen evolution reaction, and overall water-splitting reaction, respectively. *^b^* AQY, ER, and TOF represent the apparent quantum yield, evolution rate, and turnover frequency, respectively.

**Table 3:** Effective cocatalysts, possible reaction, and efficiency of water-splitting photocatalysts (Vis-light-driven or UV-light-driven) introduced in Section 3.

Photocatalyst	Cocatalyst (/wt%) *^a^*	Size /nm	Possible Reaction *^b^*	Efficiency *^c^*	Ref.
BaLa_4_Ti_4_O_15_	Au NPs (0.5)	12.3 ± 3.7	OWSR	ER (H_2_) = ~60 µmol h^−1^ (400 W Hg lamp)	[74]
	Au_10_ NC (0.1)	0.9 ± 0.2	OWSR	ER (H_2_) = ~260 µmol h^−1^ (400 W Hg lamp)	[74]
	Au_15_ NC (0.1)	1.0 ± 0.2	OWSR	ER (H_2_) = ~220 µmol h^−1^ (400 W Hg lamp)	[74]
	Au_18_ NC (0.1)	1.1 ± 0.2	OWSR	ER (H_2_) = ~210 µmol h^−1^ (400 W Hg lamp)	[74]
	Au_25_ NC (0.1)	1.2 ± 0.3	OWSR	ER (H_2_) = ~180 µmol h^−1^ (400 W Hg lamp)	[74]
	Au_39_ NC (0.1)	1.5 ± 0.3	OWSR	ER (H_2_) = ~170 µmol h^−1^ (400 W Hg lamp)	[74]
	Cr_2_O_3_/Au_25_ NC (Cr: 0.5, Au: 0.1)	1.1 ± 0.3	OWSR	ER (H_2_) = 3032 µmol h^−1^ (400 W Hg lamp)	[75]
	Au_24_Pd NC (Au: 0.1)	1.1 ± 0.2	OWSR	ER (H_2_) = ~100 µmol h^−1^ (400 W Hg lamp)	[76]
	Au_24_Pt NC (Au: 0.1)	1.1 ± 0.2	OWSR	ER (H_2_) = ~125 µmol h^−1^ (400 W Hg lamp)	[76]
	Cr_2_O_3_/ Au_24_Pt NC (Cr: 0.3, Au: 0.1)	1.3 ± 0.3	OWSR	ER (H_2_) = ~2500 µmol h^−1^ (400 W Hg lamp)	[76]
	Rh_2-*x*_Cr*_x_*O_3_ NPs (Rh: 0.09, Cr: 0.1)	1.2 ± 0.2	OWSR	ER (H_2_) = 9.9 mmol h^−1^ (400 W Hg lamp) AQY = 16% (at 270 nm)	[77]
BaTaO_2_N	Pt(IMP)/Pt(PD) NPs (Pt(IMP): 0.1, Pt(PD): 0.2)	N/A	HER	AQY = 6.8 ± 0.5% (at 420 nm)	[225]
CdS	cubic-Pd NPs	10.0	HER	ER = 814 µmol h^−1^ (>420 nm)	[223]
	octahedral- Pd NPs	7.7	HER	ER = 591 µmol h^−1^ (>420 nm)	[223]
	spherical-Pd NPs	N/A	HER	ER = 489 µmol h^−1^ (>420 nm)	[223]
	cubic-PtPd NPs (0.5)	7.3	HER	AQY = 54.0% (at 420 nm)	[224]
	octahedral-PtPd NPs (0.5)	4.7	HER	AQY = 36.6% (at 420 nm)	[224]
g-C_3_N_4_	CoAl_2_O_4_ NPs (3.0)	5–20	OER (pH = 8.0−8.5)	AQY = 0.2% (at 420 nm)	[242]
	Pt_0.5_Co_0.5_ NPs (1.0)	3–5	HER	ER = 960 µmol g^−1^ h^−1^ (≥400 nm)	[220]
	cubic-Pt NPs (0.89)	10.5	HER	ER = 20.4 µmol h^−1^ (>400 nm)	[221]
	octahedral-Pt NPs (0.90)	11.0	HER	ER = 24.7 µmol h^−1^ (>400 nm)	[221]
	spherical-Pt NPs (0.87)	9.4	HER	ER = 29.4 µmol h^−1^ (>400 nm)	[221]
GaN:ZnO	Cr_2_O_3_/Rh NPs (Cr: 1.5, Rh: 0.3)	1.5 ± 0.3	HER (pH = 4.5)	ER = ~620 µmol h^−1^ (>400 nm)	[218]
	Cr_2_O_3_/Rh NPs (Cr: 1.5, Rh: 0.3)	3.8 ± 0.8	HER (pH = 4.5)	ER = ~430 µmol h^−1^ (>400 nm)	[218]
	Cr_2_O_3_/Rh NPs (Cr: 1.5, Rh: 0.3)	6.6 ± 1.1	HER (pH = 4.5)	ER = ~260 µmol h^−1^ (>400 nm)	[218]
LaMg_1/3_Ta_2/3_O_2_N	TiOXH /SiOXHRhCrO*_x_* NPs (Cr: 0.5, Rh: 0.5)	N/A	OWSR	AQY = 0.03% (at 440 ± 30 nm)	[234]
SrTiO_3_	Cu_0.95_Pt_0.05_ NPs (1.5 mol%)	~3.0	HER	ER = 369.4 µmol h^−1^ (500 W Hg lamp)	[219]
SrTiO_3_:Al	RhZrO*_x_* NPs (Zr: 0.5, Rh: 0.1) -CoO*_x_* (0.1)	N/A	OWSR	AQY = 33 ± 4.0% (at 365 nm)	[241]
TiO_2_ (anatase)	cubic-Pd NPs (2.7)	~14	HER (pH = 3.0)	ER = ~6.0 µmol g^−1^ h^−1^ (>420 nm)	[222]
TiO_2_ (anatase)	tetrahedral-Pd NPs (2.8)	~14	HER (pH = 3.0)	ER = ~10 µmol g^−1^ h^−1^ (>420 nm)	[222]
TaON	CoO*_x_* NPs (0.15)	~2.5	OER (pH = ~8.5)	AQY = 21.2% (at 420 ± 15 nm)	[243]

*^a^* For example, Pt(IMP) and Pt(PD) NPs represent Pt NPs loaded by impregnation and photodeposition, respectively. *^b^* OER, HER, and OWSR represent the oxygen evolution reaction, hydrogen evolution reaction, and overall water-splitting reaction, respectively. *^c^* AQY, ER, and Hg represent the apparent quantum yield, evolution rate, and mercury, respectively.

**Table 4:** Effective cocatalysts, possible reaction, and efficiency of water-splitting photocatalysts (Vis-light-driven) introduced in Section 4.

Photocatalyst	Cocatalyst (/wt%) *^a^*	Size /nm	Possible Reaction *^b^*	Efficiency *^c^*	Ref.
BiVO_4_	Pt(PD) NPs -MnO*_x_*(PD) NPs (Pt: 0.1, Mn: 0.1)	Pt: 10–30 MnO*_x_*: N/A	OER (pH = 6.8)	ER = ~660 µmol h^−1^ g^−1^ (>420 nm)	[287]
CdSe NRs	Pt NPs-PdS NPs (Pt: 0.5, PdS: 0.5)	Pt: ~2–6 PdS: N/A	HER	AQY = ~ 45.0% (at 420 nm)	[289]
Cd_0.5_Zn_0.5_S:P NRs	Pt NPs-PdS NPs (Pt: N/A, Pd: N/A)	N/A	HER	AQY = 89.0% (at 420 nm)	[290]
Hollow-CdS	MoS_2_ NPs -PdS NPs (Mo: 7.5, Pd: 1.5)	N/A	HER	AQY = 20.9% (at 495 nm)	[293]
Hollow-g-C_3_N_4_	Pt/Co_3_O_4_ (1.0, 3.0)	Pt: 3.0 Co_3_O_4_: 2.0	OWSR	ER (H_2_) = 3.1 µmol h^−1^ (>300 nm) ER (O_2_) = 1.5 µmol h^−1^ (>300 nm)	[292]
SrTiO_3_:Al	Cr_2_O_3_/Rh NPs -CoOOH NPs (Cr: 0.1, Rh: 0.05, Co: 0.05)	N/A	OWSR	AQY = 96% (at 350–360 nm)	[288]
Hollow-Ta_3_N_5_	Pt NPs -IrO_2_ NPs (Pt: 1.0, IrO_2_: 0.025)	Pt: 3.0–5.0 IrO_2_: N/A	HER	ER = ~3.1 µmol h^−1^ (>400 nm)	[291]

*^a^* For example, Pt(PD) NPs represent Pt NPs loaded by photodeposition. *^b^* OER, HER, and OWSR represent the oxygen evolution reaction, hydrogen evolution reaction, and overall water-splitting reaction, respectively. *^c^* AQY, and ER, represent the apparent quantum yield, and evolution rate, respectively.
